# Development and validation of a deep learning-based microsatellite instability predictor from prostate cancer whole-slide images

**DOI:** 10.1038/s41698-024-00560-7

**Published:** 2024-04-09

**Authors:** Qiyuan Hu, Abbas A. Rizvi, Geoffery Schau, Kshitij Ingale, Yoni Muller, Rachel Baits, Sebastian Pretzer, Aïcha BenTaieb, Abigail Gordhamer, Roberto Nussenzveig, Adam Cole, Matthew O. Leavitt, Ryan D. Jones, Rohan P. Joshi, Nike Beaubier, Martin C. Stumpe, Kunal Nagpal

**Affiliations:** 1 Tempus AI, Inc, 600 W Chicago Ave #510, Chicago, IL 60654 USA; 2 Work done while at Tempus AI, Inc, 600 W Chicago Ave #510, Chicago, IL 60654 USA; 3 PathNet, Inc, 5100 Talley Rd Suite 300, Little Rock, AR 72204 USA; 4 DDx Foundation, 2889 W Ashton Blvd. Suite 300, Lehi, UT 84043 USA; 5 Lumea, 2889 Ashton Blvd #300, Lehi, UT 84043 USA

**Keywords:** Tumour biomarkers, Cancer genomics, Cancer imaging, Prostate cancer

## Abstract

Microsatellite instability-high (MSI-H) is a tumor-agnostic biomarker for immune checkpoint inhibitor therapy. However, MSI status is not routinely tested in prostate cancer, in part due to low prevalence and assay cost. As such, prediction of MSI status from hematoxylin and eosin (H&E) stained whole-slide images (WSIs) could identify prostate cancer patients most likely to benefit from confirmatory testing to evaluate their eligibility for immunotherapy and need for Lynch syndrome testing. Prostate biopsies and surgical resections from prostate cancer patients referred to our institution were analyzed. MSI status was determined by next-generation sequencing. Patients sequenced before a cutoff date formed an algorithm development set (*n* = 4015, MSI-H 1.8%) and a paired validation set (*n* = 173, MSI-H 19.7%) that consisted of two serial sections from each sample, one stained and scanned internally and the other at an external site. Patients sequenced after the cutoff date formed a temporally independent validation set (*n* = 1350, MSI-H 2.3%). Attention-based multiple instance learning models were trained to predict MSI-H from H&E WSIs. The predictor achieved area under the receiver operating characteristic curve values of 0.78 (95% CI [0.69–0.86]), 0.72 (95% CI [0.63–0.81]), and 0.72 (95% CI [0.62–0.82]) on the internally prepared, externally prepared, and temporal validation sets, respectively, showing effective predictability and generalization to both external staining/scanning processes and temporally independent samples. While MSI-H status is significantly correlated with Gleason score, the model remained predictive within each Gleason score subgroup.

## Introduction

Prostate cancer is the second most common cancer in the United States, with approximately one in eight men receiving a prostate cancer diagnosis in their lifetime, and represents the fifth most common cause of cancer mortality^1^. Despite an increasing number of targeted and immunotherapy treatment options in cancer overall^2^, the use of these modalities has lagged in prostate cancer. Neither comprehensive next-generation sequencing (NGS) testing nor mismatch repair (MMR) protein immunohistochemistry (IHC) is standard of care and testing rates are low, since they typically cost several hundred to several thousand dollars and utilize several slides of tissue^3^. Testing frequency in prostate cancer is in contrast with colorectal cancer, for example, where the higher prevalence of mismatch repair deficient (dMMR) and microsatellite instability-high (MSI-H) has allowed testing to become standard^4^.

MSI-H is a biomarker caused by a deficiency in DNA mismatch repair and is associated with response to immune checkpoint inhibitor therapy. While there is not currently a prostate cancer specific approval, pembrolizumab, a PD-1 inhibitor, has a tumor-agnostic approval for use in unresectable or metastatic MSI-H or dMMR solid tumors. However, NCCN Guidelines currently recommend MMR/microsatellite instability (MSI) testing only for metastatic castration-resistant prostate cancer^5^, ESMO and PanAsia guidelines state that testing may be considered for this population^6^. Collectively, these result in significant metastatic or high-risk regional cancer patients that are not outwardly recommended for testing under current guidelines. In prostate cancer, MSI-H is uncommon and has been reported at only 2–3% prevalence^7,8^. However, overall response rates to immune checkpoint inhibitors of 25–60%, including durable responses, have been reported across several small studies in this subgroup of patients^7,9,10^. Biomarker-unselected prostate cancer populations have shown limited benefit from immune checkpoint inhibitors, highlighting the critical importance of biomarker testing for MSI-H or dMMR for prostate cancer immunotherapy to enrich for responders despite its low prevalence^11–13^.

While MSI testing is not routinely performed for prostate cancer patients, prostate cancer diagnosis nearly always involves a tissue biopsy with hematoxylin and eosin (H&E)-stained slides and residual formalin-fixed paraffin-embedded (FFPE) tissue, which can be used for NGS or IHC stains. The H&E stained slides are increasingly being digitized as whole slide images (WSIs) to assist pathology workflows and for archival purposes. Therefore, predicting MSI status from H&E WSIs is potentially impactful for identifying patients who are likely MSI-H and may benefit from confirmatory testing for their MSI status to evaluate their eligibility for immunotherapy and/or Lynch syndrome testing.

The application of machine learning on WSIs has been studied for predicting MSI-H in colorectal and gastric cancers^14–16^. However, MSI prediction in prostate cancer has been less well studied, with lower prevalence and lack of testing in the standard of care posing challenges to collecting sufficient positive samples. This absence of testing in standard care also creates an unmet need to identify MSI-H/dMMR tumors, and H&E-based machine learning models could assist in narrowing down the population to be tested so that it becomes feasible to do so. Moreover, the generalizability of histopathology machine learning algorithms across multi-site staining and scanning characteristics remains a significant challenge, and validating algorithm performance across external pre-analytic characteristics remains important for algorithm utility^17^.

In this study, we developed a machine-learning model to predict MSI-H from a large, real-world prostate cancer cohort containing WSIs, clinical data, molecular testing results, and IHC assay results. We directly validated the generalizability of the predictor to stain and scanner characteristics by evaluating performance on an externally prepared dataset composed of a serial section of each slide from the internal validation set but stained at a different site and scanned using a different scanner model. We also validated the model’s generalizability to a temporally independent internal validation cohort. We conducted several subgroup analyses, including procedure types and Gleason score subgroups. The predictor demonstrated high effectiveness in identifying MSI-H from WSIs and has the potential to identify prostate cancer patients most likely to benefit from confirmatory testing for their MSI status.

## Results

### Cohort characteristics

Table 1 and Supplementary Table 1 present the patient characteristics of the cohorts. A multivariate logistic regression model that predicts MSI status based on the clinical and demographic variables of the cohort shows that Gleason score, sample collection date, and tumor mutational burden (TMB) have statistically significant coefficients (Supplementary Table 2). Other variables that showed significant univariate correlation with MSI status in the cohort tables did not remain significant in multivariate analysis. Higher Gleason scores are associated with greater MSI-H prevalence, ranging from 0.6% amongst Gleason 7 cases to 8.5% amongst Gleason 10 cases. No other significant correlations were found between MSI status and clinical or demographic variables.

**Table 1:** Patient characteristics in data cohorts

	Overall			Training set		Paired validation set		Temporal validation set	
Variable	MSI-H, *N* = 138^a^	MSS, *N* = 5400^a^	*p*-value^b^	MSI-H, *N* = 73^a^	MSS, *N* = 3942^a^	MSI-H, *N* = 34^a^	MSS, *N* = 139^a^	MSI-H, *N* = 31^a^	MSS, *N* = 1319^a^
Age at collection date	71 (65, 76)	66 (60, 73)	<0.001	71 (64, 76)	66 (60, 72)	71 (65, 76)	68 (61, 73)	69 (63, 77)	68 (61, 74)
Unknown	14	892	–	6	524	1	16	7	352
Race			0.5						
Asian (%)	3 (4.2)	67 (2.7)	–	1 (2.9)	56 (2.9)	1 (4.8)	1 (1.3)	1 (6.7)	10 (2.0)
Black or African American (%)	11 (15)	472 (19)	–	2 (5.7)	355 (19)	4 (19)	11 (15)	5 (33)	106 (21)
White (%)	57 (80)	1942 (78)	–	32 (91)	1494 (78)	16 (76)	63 (84)	9 (60)	385 (77)
Unknown	67	2919	–	38	2037	13	64	16	818
Histology			0.6						
Adenocarcinoma (%)	136 (99)	5315 (98)	–	71 (97)	3874 (98)	34 (100)	135 (97)	31 (100)	1306 (99)
Carcinoma (%)	1 (0.7)	17 (0.3)	–	1 (1.4)	15 (0.4)	0 (0)	1 (0.7)	0 (0)	1 (<0.1)
Neuroendocrine (%)	1 (0.7)	51 (0.9)	–	1 (1.4)	40 (1.0)	0 (0)	1 (0.7)	0 (0)	10 (0.8)
Sarcoma (%)	0 (0)	3 (<0.1)	–	0 (0)	3 (<0.1)	–	–	–	–
Small cell carcinoma (%)	0 (0)	14 (0.3)	–	0 (0)	10 (0.3)	0 (0)	2 (1.4)	0 (0)	2 (0.2)
Total gleason			<0.001						
7 (%)	5 (4.3)	878 (21)	–	3 (4.8)	692 (22)	0 (0)	9 (8.1)	2 (8.7)	177 (20)
8 (%)	18 (15)	832 (20)	–	9 (15)	617 (20)	3 (9.4)	11 (9.9)	6 (26)	204 (22)
9 (%)	61 (52)	2097 (50)	–	33 (53)	1605 (51)	17 (53)	51 (46)	11 (48)	441 (49)
10 (%)	33 (28)	370 (8.9)	–	17 (27)	245 (7.8)	12 (38)	40 (36)	4 (17)	85 (9.4)
Unknown	21	1223	–	11	783	2	28	8	412
Procedure type			0.5						
Ambiguous biopsy (%)	18 (13)	578 (11)	–	11 (15)	431 (12)	3 (9.1)	12 (8.9)	4 (13)	135 (10)
Core needle biopsy (%)	80 (59)	2910 (56)	–	42 (58)	2009 (54)	18 (55)	77 (57)	20 (65)	824 (63)
Resection + excisional (%)	38 (28)	1691 (33)	–	19 (26)	1293 (35)	12 (36)	46 (34)	7 (23)	352 (27)
Unknown	2	221	–	1	209	1	4	0	8

^a^Median (IQR); *n* (%).

^b^Wilcoxon rank-sum test; Fisher’s exact test; Pearson’s *χ*^2^ test.

Table 2 characterizes the MMR results where IHC stains were also available. For MSI-H cases, MSH2/MSH6 absence was the most common abnormal MMR staining pattern, occurring in 32/38 (84.2%) of cases, followed by four cases of MLH1/PMS2 absence (10.5%), and one case of PMS2-only absence (2.6%). One case had no MMR protein loss detected (2.6%), but NGS detected an MSH6 missense mutation, E1193K, which has previously been determined to impair heterodimerization with MSH2 and resulting MMR capability^18^. This distribution is consistent with other studies on dMMR in prostate cancer and different from other cancer types such as colorectal and endometrial cancers ^7,19–23^.

**Table 2:** Distribution of mismatch repair (MMR) immunohistochemical (IHC) stain findings for prostate cancer cases

IHC staining pattern	Number of MSI-H samples	Number of MSS samples
MSH2/MSH6 Loss	32	6
MLH1/PMS2 Loss	4	0
MSH6 loss only	0	3
PMS2 loss only	1	2
All present	1^a^	1345
MMR IHCs not available	100	4044

^a^For the one MSI-H case where all MMR staining patterns were present, NGS detected an MSH6 missense mutation (E1193K).

### Model performance

An attention-based multiple instance learning network was trained on tiles randomly sampled from H&E WSI tissue regions to predict MSI-H. The MSI-H predictor achieved area under receiver operating characteristic curve (AUC) values with confidence intervals (CIs) of 0.78 (95% CI [0.69–0.86]), 0.72 (95% CI [0.63–0.81]), and 0.72 (95% CI [0.62–0.82]) on internally stained and scanned, externally stained and scanned, and temporal validation sets, respectively (Fig. 3). The difference in AUC between each pair of validation sets did not show statistical significance: ΔAUC = 0.06 (95% CI [−0.05, 0.17]) between the paired validation sets and ΔAUC = 0.06 (95% CI [−0.08, 0.20]) between the internal paired validation set and the internal temporal validation set. A significant correlation between prediction scores on the paired internally and externally stained and scanned serial sections was observed (*R* = 0.85, 95% CI [0.77, 0.91], Supplementary Fig. 1). At an example operating point of 50% sensitivity, the MSI-H predictor had a specificity of 86.8% (95% CI [59.9%, 95.7%]), a positive predictive value (PPV) of 7.9% (95% CI [2.7%, 23.2%]), and a negative predictive value (NPV) of 98.6% (95% CI [97.9%, 99.1%]) on the temporal validation set. The PPV is notably higher than the underlying MSI-H prevalence of 2.3% in our cohort and the reported 2–3% in the literature. A review of the high-attention tiles suggests the predictor focuses on dense tumor regions in making its determination, while its low-attention tiles largely comprise tiles with stroma and whitespace (Supplementary Fig. 2).

We assessed performance within subgroups on a pooled validation set combining the internally stained and scanned images in the paired validation and the temporal validation sets (Fig. 4). The ROC curves and the violin plots of prediction scores show that the model remained predictive of MSI-H status within each Gleason score and procedure type subgroup. AUC trended higher in the Gleason scores 7–8 subgroup (AUC = 0.80, 95% CI [0.66, 0.94]). In the Gleason scores 9–10 subgroup, where MSI-H prevalence is the highest, patients are classified as high-risk, and the need for therapy is often significant, the AUC was also encouraging (AUC = 0.72, 95% CI [0.64, 0.81]), and the distributions of prediction scores for MSI-H and microsatellite-stable (MSS) patients were significantly different. Performance within surgical resections trended higher than within biopsies (AUC = 0.86, 95% CI [0.77, 0.95] vs. AUC = 0.73, 95% CI [0.65, 0.80]), and the distributions of prediction scores for MSI-H and MSS patients were significantly different in both subgroups, potentially owing to larger tissue context and reduced frequency of biopsy-related artifacts. Subgroup analysis within each validation set shows qualitatively similar trends but did not have adequate statistical power to assess significance in several subgroups owing to smaller sample sizes (Supplementary Fig. 3).

Additional subgroup analyses showed that the algorithm performance remained robust on small specimens with tissue area in the lowest quartile (AUC = 0.76, 95% CI [0.61, 0.92]) and trended slightly lower on samples with tumor purity in the lowest quartile (AUC = 0.71, 95% CI [0.61, 0.83]) (Supplementary Fig. 4a). The tissue area simulation experiment showed that the model performance remained robust down to bag sizes of 50–100 tiles, corresponding to 0.6–1.3 mm^2^ of sampled tissue area, which is the 0.01 percentile in our dataset and is much smaller than a core needle biopsy. The tumor purity simulation experiment showed that model performance remained unchanged at high tumor percentages (70% tumor tiles and above) and decreased somewhat at low tumor percentages, but remained significantly predictive (Supplementary Fig. 4b). Note that a bag consisting of 70% tumor tiles and 30% stroma tiles is equivalent to a tissue area of less than 70% tumor purity, since we used tumor region annotation, rather than cell-level annotation.

Finally, the data titration experiment showed that model performance on the validation sets increased as a larger fraction of training data was used, and the model performance may yet improve with additional training data (Supplementary Fig. 5).

## Discussion

In this study, we developed a deep learning predictor of MSI status using a large, real-world cohort of H&E whole slide images and corresponding molecular testing results and evaluated its generalizability to externally stained and scanned slides and to a temporally independent validation cohort. The predictor achieved high performance for a screening algorithm and demonstrated significant discriminative ability on both the externally stained and scanned images and the temporal validation set. Given the predictor’s effectiveness and generalizability, the ubiquity and increasing digitization of H&E slides in prostate cancer diagnoses, and the lack of routine testing for MSI in prostate cancer, we anticipate that our algorithm could be used to direct testing and find patients eligible for targeted therapies who otherwise may have been missed.

For patients determined to be MSI-H via confirmatory testing, the clinical implications are significant, including potential eligibility to receive pembrolizumab, which has a tumor-agnostic indication in MSI-H/dMMR tumors and reported response rates of 25–60%^7,9–11^. Other immunotherapies may also be effective, with evidence of encouraging response rates to nivolumab, a PD-1 inhibitor in a Phase II clinical trial^24^. These findings show that our MSI predictor is potentially impactful on patient outcomes.

Furthermore, our analysis revealed a notable pattern of concurrent loss of MSH2 and MSH6 expression in cases of MMR deficiency, which may indicate an increased likelihood of Lynch syndrome^25^. Lynch syndrome results from defective mismatch repair mechanisms caused by germline mutations in MMR, which significantly raises the lifetime cancer risk^25^. Consequently, the detection and monitoring of MSI-H are crucial, not only for patient treatment but also for potential enhanced surveillance protocols for their families, considering heritable cancer risks^26^. Other studies have reported Lynch syndrome prevalence in prostate cancer at 0.6–0.8%, which would be a notable fraction of all MSI-H prostate cancers^7,27^. Given that the Tempus xT assay is primarily used for assessing somatic mutations, with detection of potential germline genes associated with Lynch syndrome possible via sequencing of matched normal controls^28^, precise quantitation of Lynch syndrome in the study cohort is not feasible. However, potential germline findings were present in a substantial fraction of the cohort (see Supplementary Information)^29^, consistent with the above suggestion that Lynch syndrome is present in a meaningful subset of MSI-H prostate cancers and furthering the importance of MSI-H detection.

Gleason score is an important prognostic measure in prostate cancer that is often used in patient risk stratification^30^. Subgroup analyses showed that the model remained predictive within Gleason score subgroups, including scores of 9–10, where the impact of this algorithm may be the greatest. Patients exhibiting Gleason scores of 9–10 (Grade Group 5) have significantly worse prognosis than other prostate cancer patients^31^, are minimally considered stage IIIC independent of metastatic status^32^, and correspondingly tend to receive aggressive treatment including hormonal and radiation therapy. The MSI-H prevalence is also greatest amongst these patients in our study, and similar associations have been noted in other studies^33^. While MSI-H has been associated with favorable prognosis in other cancer types, the prognostic significance of MSI in high Gleason score prostate cancer is not yet fully understood, and treatment selection for these patients remains a significant need. Given the greater prevalence and significant clinical need for treatment in high-grade prostate cancers, we anticipate the predictor’s utility and urgency of MSI-H confirmatory testing may be the greatest in this subgroup.

Compared with the model performance on the internally stained and scanned slides, the performance dropped slightly on the externally stained and scanned slides in the paired validation set. While not statistically significant, the performance difference could be attributed to differences in staining and scanning protocols, a well-known challenge for the generalizability of deep learning algorithms in digital pathology. Subgroup analyses showed, as expected, that Philips UFS scans contributed more to the difference between internal and external scans than the Leica GT450 scans, given that the externally prepared slides were scanned on a Leica AT2 scanner. We employed robustness measures, such as the International Color Consortium (ICC) profile transformation and color augmentation during model training, to reduce domain shift and increase model generalizability. Additionally, although all patients were sequenced at Tempus, 37% of H&E slides in our cohort were prepared externally at other laboratories using different staining protocols and scanners, mimicking diverse data in a multi-institutional study and adding confidence to the generalizability of our model.

While not statistically significant, the model performance is also slightly lower on the temporally independent validation set than the internal slides in the paired validation set. The two validation sets have some different characteristics that may contribute to some differences in performance. For example, samples submitted for sequencing at Tempus can either be submitted as a block, for which Tempus cuts and stains an H&E slide, or a set of pre-cut slides, where one of the slides is already stained externally with H&E. The paired internal set contains only Tempus-stained H&Es, while the temporal validation set contains both Tempus-stained H&Es and externally stained H&Es. Additional difference could include temporal drifts in data distribution, such as shifts in patient population or staining techniques.

There are several limitations to our work. First, the performance and generalizability that our model achieved were constrained by the limited number of MSI-H cases in our cohort and may benefit from additional data. Also, the application of this algorithm is currently restricted to primary tumor specimens, and the extension to metastatic site specimens can be studied in future work. Moreover, performance was evaluated on one slide per case, while several slides are typically produced for each case during typical pathology workflows. Future work is needed to study the selection of optimal slides for the predictor to analyze for a given case. While the model demonstrated potential utility on small biopsy specimens, the subgroup analysis and simulation experiments showed trends toward algorithm performance degradation at low tumor purities. If this algorithm were to be deployed, one workflow might include having a pathologist roughly outline a high tumor purity region or identify the highest tumor purity slide available from case-upon-case digitization. Future work could simplify the workflow via the utilization of an automatic tumor detector.

Furthermore, as a screening test to encourage or triage for confirmatory testing, the clinical operating point would depend on the clinician’s judgment for the trade-off between the costs and benefits of confirmatory testing. While we cannot recommend an operating point or acceptance criteria for all patients’ situations, we believe colorectal cancer that has a universal MMR/MSI testing recommendation could serve as a useful comparison. The underlying prevalence is ~13% for colorectal cancer and can serve as a target PPV for operating point determination^34^. Assuming a real-world prevalence of 3.1% in prostate cancer, a post-test probability of ≥13% would require a specificity of ≥ 93% at 30% sensitivity, which is achievable using our model as shown in Fig. 3c.

Finally, although we constructed a paired validation set to evaluate model generalizability to externally stained and scanned slides, our real-world dataset has inherent biases stemming from the retrospective inclusion of only patients who underwent sequencing and the utilization of single-institution sequencing results. As such, a prospective, multi-institution evaluation of the MSI-H predictor is warranted prior to the algorithm’s use in clinical practice.

## Methods

### Study design and participants

In this retrospective, diagnostic study, we sampled consecutive prostate cancer patients who were sequenced at Tempus Labs (Chicago, IL, USA) from October 2017 to February 2023 and whose WSIs of prostate biopsies or surgical resections were available. Each case included clinical characteristics, molecular profiles, and digitized WSIs. This study was conducted on de-identified health information subject to an IRB exempt determination (Advarra Pro00072742) and did not involve human subjects research.

**Fig. 1:**
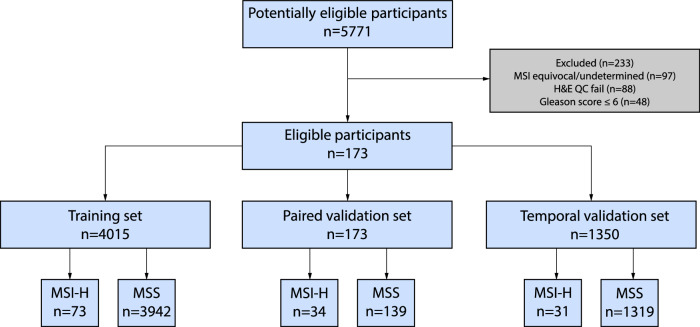
STARD diagram of the study.

About 25% of cases have MMR protein IHC. All samples were digitized with either a Philips UFS scanner or an Aperio GT 450 scanner, and 37% of samples were stained at external laboratories. MSI-H/MSS status was determined using DNA NGS. The NGS assay used in this study, Tempus xT, is a laboratory-developed test used for tumor profiling of solid malignant neoplasms. The test for MSI status in the xT panel, when compared with results of IHC staining, has 90.5% PPA, and 98.2% NPA for non-colorectal and non-endometrial cancers^35,36^. Samples with equivocal or undetermined MSI status (*n* = 97), samples that failed quality control by pathologists (*n* = 88), and samples with Gleason scores of less than 7 (*n* = 48) were excluded (Fig. 1). Reasons for equivocal or undetermined MSI status predominantly result from insufficient tumor purity for the MSI call in NGS, but may also include sequencing of insufficient depth over enough of the assayed microsatellite loci to prevent the prediction from reaching statistical significance, or in rare cases (*n* = 2) multiple tests for the same patient returning conflicting results.

**Fig. 2:**
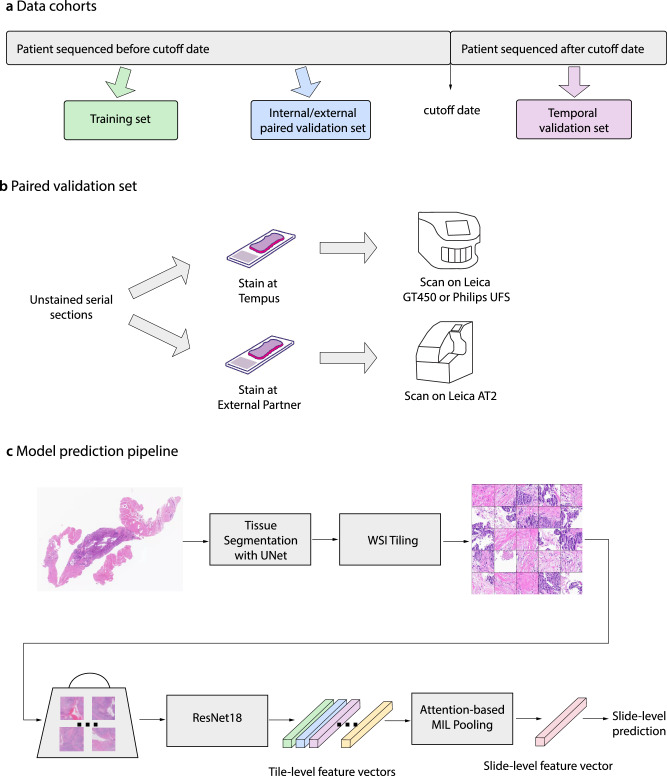
Overview of study design. Schematic representation of **a** data cohorts, **b** paired validation set, and **c** model pipeline.

A cutoff sequencing date in July 2022 was selected to split the cohort into two temporally independent subsets. Patients sequenced before a cutoff date (*n* = 4188, MSI-H 2.6%), formed the training set and a paired validation set. The training set (*n* = 4015, MSI-H 1.8%) was used for model development and the paired set (*n* = 173, MSI-H 19.7%) to directly evaluate stain and scanner generalizability. The paired validation set was composed of two serial sections from each sample, one of which was stained and scanned internally and another stained and scanned at an external site, TruCore Pathology (Little Rock, AR), using an Aperio AT2 scanner. This set was constructed by randomly sampling 36 MSI-H cases where an unstained serial section was available for study use, and correspondingly sampling 144 MSS cases with matched Gleason score and procedure type distribution as the selected MSI-H samples prior to quality control exclusions. Patients sequenced after the cutoff date formed the temporal validation set (*n* = 1350, MSI-H 2.3%), which was used to evaluate model generalizability on temporally independent data. The design of the data cohorts is illustrated in Fig. 2a, b. Validation sets were held out from model development and were only used for evaluation and reporting of metrics.

### Model development

Tissue and marker regions were first identified on WSIs using a previously developed U-Net model^37^. Subsequently, tiles of size 256 × 256 pixels at 20x magnifications were generated from WSIs. Tiles predicted to not contain tissue or to contain markers were excluded. ICC profile transformations^38^ were applied to correct the color discrepancies between Leica GT450, Leica AT2, and Philips UFS scanners.

An attention-based multiple instance learning model similar to Ilse et al. was trained using these images^39^, as illustrated in Fig. 2c. This model architecture is suitable for the task because it allows for in-depth tile-level feature analysis while using slide-level labels of MSI status in a weakly supervised training scheme. The model accepts a “bag of tiles” as input. An ImageNet pre-trained ResNet18 model was used as a feature extraction module for each tile^40^, while the attention module was used to identify tiles with high diagnostic relevance and aggregate information from all tiles in the bag to make a slide-level prediction. The entire model was trained end-to-end using Adam optimizer and weighted cross-entropy loss where weights were assigned according to the class prevalence^41^. In each epoch during training, 200 tiles were randomly sampled to form a bag. The effective batch size was 32 during training, split across four NVIDIA A100 GPUs. At inference time, the bag size was increased to 1600 with a batch size of 1 on one NVIDIA A100 GPU. Tile sampling was performed without replacement, and if a slide had fewer tiles than the bag size, all tiles in the slide were used. Tiles were normalized with the mean and standard deviation of a reference set of H&E images. For data augmentation during training, tiles were randomly cropped to 224 × 224, randomly rotated by multiples of 90 degrees, randomly flipped, and randomly applied with color jittering.

5-fold cross-validation within the training set was used to perform hyperparameter tuning to select the learning rate, weight decay, dropout rate, patience and minimum delta for early stopping, input image magnification, and color augmentation parameters (see Supplementary Information for detailed information). Data splitting for creating cross-validation folds was done such that MSI status and potential confounding variables, such as scanner type, procedure type, and Gleason score were represented equally in each fold. Once the final hyperparameters were selected, the MSI-H predictor was composed by averaging the predictions across the five models trained via cross-validation using the selected hyperparameters. This predictor was finally evaluated on three validation sets: the paired validation set with enriched MSI-H prevalence composed of internally and externally stained and scanned serial sections for each sample, as well as the temporal validation set to evaluate temporal generalizability.

### Evaluation

AUC was used as the main metric to evaluate classification performance. Sensitivity, specificity, PPV, and NPV were also reported to assess model performance at various target sensitivity levels. The Pearson correlation coefficient, *R*, was used to evaluate the correlation between predictions on internally and externally stained and scanned images in the paired validation set. The 95% CIs of all metrics were calculated by bootstrapping the prediction scores with 1000 bootstrap samples.

To assess the robustness of model performance across Gleason score and procedure type, subgroup analyses were performed on the pooled internal validation set, which combined the temporal validation set and the internally stained and scanned slides from the paired validation set.

Additional analyses were performed on the pooled internal validation set to assess the robustness of model performance in two challenging subgroups: specimens with small tissue area or low tumor purity. Samples with tissue area or tumor purity in the lowest quartile in the validation set formed these subgroups, corresponding to ≤9.35 mm^2^ tissue area and ≤50% tumor purity. A simulation experiment was also performed to establish the model’s limit of detection on tissue area. Model inference was run with different bag sizes, randomly sampling 3, 6, 12, 25, 50, 100, 200, 400, and 800 tiles from each slide in the validation set. Another simulation experiment was run to study the influence of tumor purity on model performance. We collected manual annotations of tumor areas for 35 MSI-H and 35 MSS slides from pathologists and simulated tumor percentages ranging from 30% to 100% by sampling different proportions of tumor tiles and stroma tiles when composing bags of tiles. The 5-fold cross-validation ensemble AUC was calculated and compared in these experiments, which were repeated 10 times with different random seeds.

**Fig. 3:**
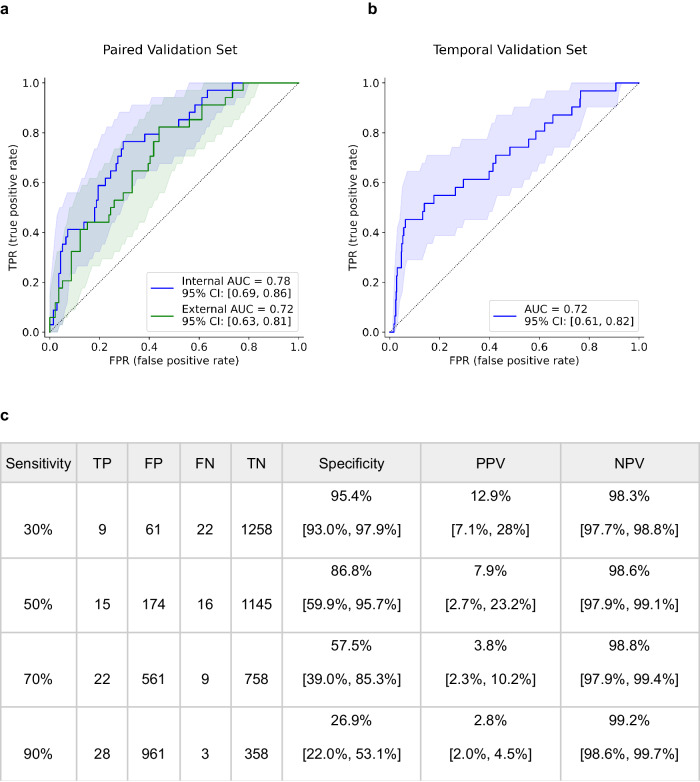
Performance of the MSI-H predictor. Receiver operating characteristic (ROC) curve for the MSI-H predictor on **a** the paired validation set and **b** the temporal validation set, and **c** a table of performance metrics and their 95% confidence intervals at various target sensitivities on the temporal validation set.

**Fig. 4:**
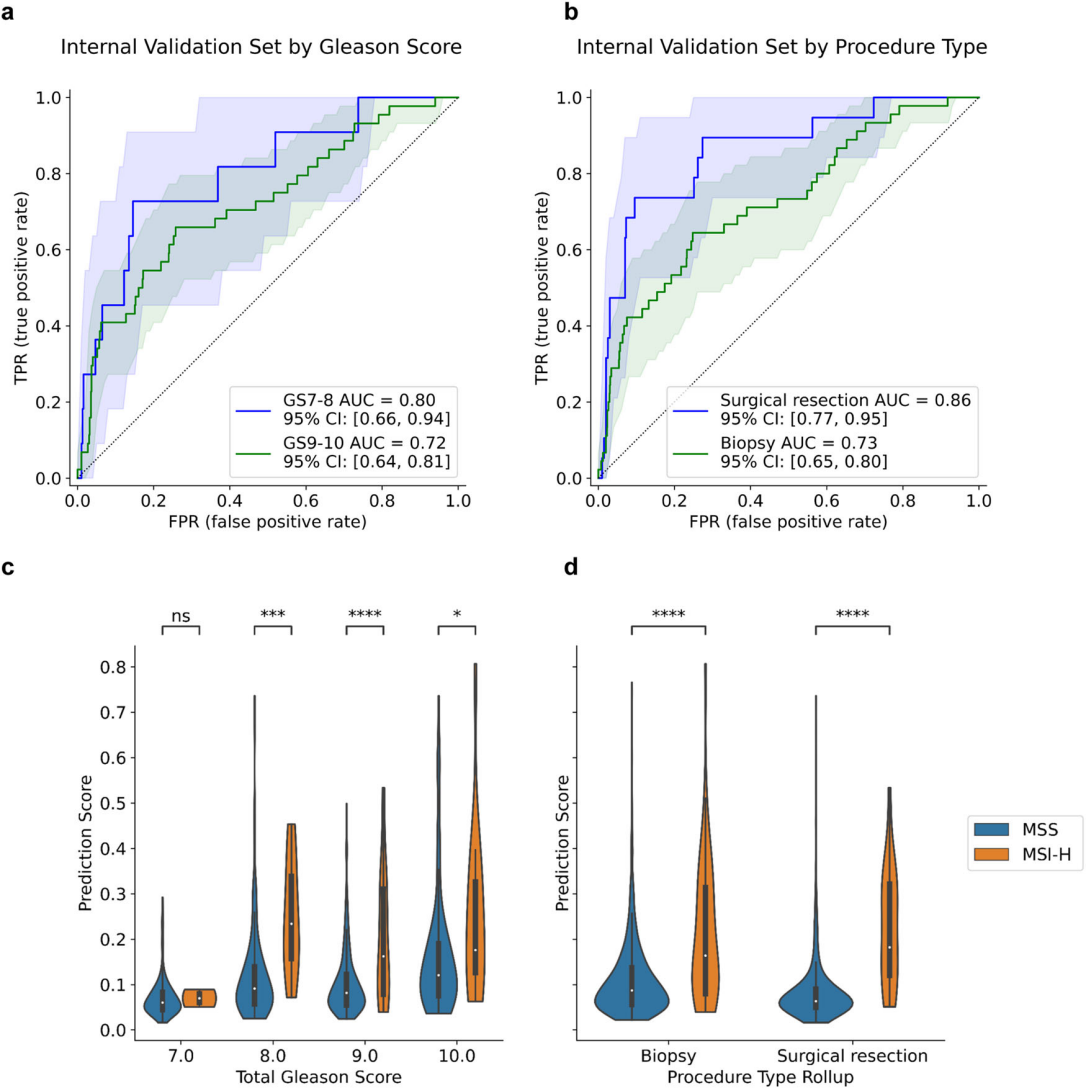
Performance of the MSI-H predictor on Gleason score and procedure type subgroups. Receiver operating characteristic (ROC) curves (**a**, **b**) and violin plots (**c**, **d**) of prediction scores for Gleason score (**a**, **c**) and procedure type (**b**, **d**) clinical subgroups in the pooled validation set that combines the internal scans of the paired validation set and the temporal validation set. The shared areas represent the 95% confidence intervals of the ROC curves. *P*-value annotation legend: ns: *p* ≤ 1, *: 0.01 < *p* ≤ 0.05, **: 0.001 < *p* ≤ 0.01, ***: 0.0001 < *p* ≤ 0.001, ****: *p* ≤ 0.0001.

We evaluated how the amount of training data would affect the algorithm performance in a data titration experiment. The training set was consecutively sub-sampled without replacement to 80%, 60%, 40%, and 20% of the original size, stratified by MSI status. A model was trained on each of these subsets using the same hyperparameters and configurations as the original model developed on the full training set, and model performance was analyzed to investigate the impact of sample size available for model development (Figs. 3 and 4).

Finally, attention heatmaps as well as high- and low-attention tiles from samples in the validation sets were visualized to inspect regions that the model deemed important in making slide-level predictions. Pathologists reviewed randomly sampled high- and low-attention tiles from MSI-H and MSS slides as a sanity check for the model behavior and to identify prominent features. A U.S. board-certified pathologist provided their blinded assessments of 60 slides (15 in each of the true positive, true negative, false positive, false negative categories) on lymphocytes within and around the tumor, predominant growth pattern, and histology of the highest Gleason pattern on each slide.

### Statistical analysis

For analyzing variable correlations with MSI status in the cohort characteristics tables, the Wilcoxon rank-sum test was used for continuous variables, the Pearson’s Chi-square test was used for categorical variables when no expected cell count was less than five, and the Fisher test was used for categorical variables when any expected cell count was less than five. The two-sided Mann–Whitney *U* test was used to compare the prediction score distributions between MSI-H and MSS samples in the subgroup analysis. A *p* < 0.05 was considered to indicate a statistically significant difference. All statistical analyses were done using R 4.2.3 (package: gtsummary 1.7.1) and Python version 3.7.12 (package: statannotations version 0.5.0) ^42,43^.

### Reporting summary

Further information on research design is available in the Nature Research Reporting Summary linked to this article.

### Supplementary information

Reporting summary

Supplementary Information

## Data Availability

Data used in the research were collected in a real-world healthcare setting and are subject to controlled access for privacy and proprietary reasons. When possible, derived data supporting the findings of this study have been made available within the paper and its supplementary materials.

## Code availability

The underlying code for this study is not publicly available but may be made available to qualified researchers upon reasonable request from the corresponding author.
