# Supplementary material for: Development and validation of a deep learning-based microsatellite instability predictor from prostate cancer whole-slide images
Source: NPJ Precis Oncol. 2024 Apr 9;8:88. doi: 10.1038/s41698-024-00560-7 (PMC11004110; doi:10.1038/s41698-024-00560-7)
Supplement: Supplementary file 1 — Reporting summary [file 41698_2024_560_MOESM1_ESM.pdf]

nature portfolio

Corresponding author(s): Kunal Nagpal

Last updated by author(s): Dec 11, 2023

Reporting Summary

Nature Portfolio wishes to improve the reproducibility of the work that we publish. This form provides structure for consistency and transparency in reporting. For further information on Nature Portfolio policies, see our Editorial Policies and the Editorial Policy Checklist.

Statistics

For all statistical analyses, confirm that the following items are present in the figure legend, table legend, main text, or Methods section.

n/a	Confirmed	
☐	☒	The exact sample size (*n*) for each experimental group/condition, given as a discrete number and unit of measurement
☐	☒	A statement on whether measurements were taken from distinct samples or whether the same sample was measured repeatedly
☐	☒	The statistical test(s) used AND whether they are one- or two-sided<br>*Only common tests should be described solely by name; describe more complex techniques in the Methods section.*
☒	☐	A description of all covariates tested
☒	☐	A description of any assumptions or corrections, such as tests of normality and adjustment for multiple comparisons
☐	☒	A full description of the statistical parameters including central tendency (e.g. means) or other basic estimates (e.g. regression coefficient) AND variation (e.g. standard deviation) or associated estimates of uncertainty (e.g. confidence intervals)
☐	☒	For null hypothesis testing, the test statistic (e.g. *F*, *t*, *r*) with confidence intervals, effect sizes, degrees of freedom and *P* value noted<br>*Give P values as exact values whenever suitable.*
☒	☐	For Bayesian analysis, information on the choice of priors and Markov chain Monte Carlo settings
☒	☐	For hierarchical and complex designs, identification of the appropriate level for tests and full reporting of outcomes
☒	☐	Estimates of effect sizes (e.g. Cohen's *d*, Pearson's *r*), indicating how they were calculated

Our web collection on statistics for biologists contains articles on many of the points above.

Software and code

Policy information about availability of computer code

Data collection	The underlying code for this study is not publicly available.
Data analysis	All statistical analyses were done using R 4.2.3 (package: gtsummary 1.7.1) and Python version 3.7.12 (package: statannotations version 0.5.0).

For manuscripts utilizing custom algorithms or software that are central to the research but not yet described in published literature, software must be made available to editors and reviewers. We strongly encourage code deposition in a community repository (e.g. GitHub). See the Nature Portfolio guidelines for submitting code & software for further information.

Data

Policy information about availability of data

All manuscripts must include a data availability statement. This statement should provide the following information, where applicable:

- Accession codes, unique identifiers, or web links for publicly available datasets
- A description of any restrictions on data availability
- For clinical datasets or third party data, please ensure that the statement adheres to our policy

Data used in the research were collected in a real-world health care setting and are subject to controlled access for privacy and proprietary reasons. When possible, derived data supporting the findings of this study have been made available within the paper and its supplementary materials.

Research involving human participants, their data, or biological material

Policy information about studies with human participants or human data. See also policy information about sex, gender (identity/presentation), and sexual orientation and race, ethnicity and racism.

Reporting on sex and gender	*Use the terms sex (biological attribute) and gender (shaped by social and cultural circumstances) carefully in order to avoid confusing both terms. Indicate if findings apply to only one sex or gender; describe whether sex and gender were considered in study design; whether sex and/or gender was determined based on self-reporting or assigned and methods used. Provide in the source data disaggregated sex and gender data, where this information has been collected, and if consent has been obtained for sharing of individual-level data; provide overall numbers in this Reporting Summary. Please state if this information has not been collected. Report sex- and gender-based analyses where performed, justify reasons for lack of sex- and gender-based analysis.*
Reporting on race, ethnicity, or other socially relevant groupings	*Please specify the socially constructed or socially relevant categorization variable(s) used in your manuscript and explain why they were used. Please note that such variables should not be used as proxies for other socially constructed/relevant variables (for example, race or ethnicity should not be used as a proxy for socioeconomic status). Provide clear definitions of the relevant terms used, how they were provided (by the participants/respondents, the researchers, or third parties), and the method(s) used to classify people into the different categories (e.g. self-report, census or administrative data, social media data, etc.) Please provide details about how you controlled for confounding variables in your analyses.*
Population characteristics	*Describe the covariate-relevant population characteristics of the human research participants (e.g. age, genotypic information, past and current diagnosis and treatment categories). If you filled out the behavioural & social sciences study design questions and have nothing to add here, write "See above."*
Recruitment	*Describe how participants were recruited. Outline any potential self-selection bias or other biases that may be present and how these are likely to impact results.*
Ethics oversight	*Identify the organization(s) that approved the study protocol.*

Note that full information on the approval of the study protocol must also be provided in the manuscript.

Field-specific reporting

Please select the one below that is the best fit for your research. If you are not sure, read the appropriate sections before making your selection.

☒ Life sciences ☐ Behavioural & social sciences ☐ Ecological, evolutionary & environmental sciences

For a reference copy of the document with all sections, see nature.com/documents/nr-reporting-summary-flat.pdf

Life sciences study design

All studies must disclose on these points even when the disclosure is negative.

Sample size	For all cohorts, sample sizes were determined based on the maximum number of samples available which respect the inclusion criteria detailed below.
Data exclusions	Inclusion criteria for all cohorts were as follows: available hematoxylin and eosin (H&E) stained whole-slide images of prostate biopsies or surgical resections from primary tumor that passed quality control, available unequivocal MSI status, Gleason score >= 7
Replication	The results presented in the paper were generated by reproducible software.
Randomization	Patients of the development cohort were randomly divided into 5 folds for cross-validation, stratified by their MSI status and other potential confounding variables. The paired validation set was constructed by randomly sampling MSI-H cases where an unstained serial section was available for study use, and randomly sampling MSS cases with matched Gleason score and procedure type distribution as the selected MSI-H samples. No randomization was applied for the temporal validation set.
Blinding	There was no patient overlap between the model development set and validation sets, so the algorithm was blinded to each patient's MSI status in the validation sets.<br>Regarding pathologist assessment for model interpretability, pathologists were independently assigned slides review and were blinded to the model predictions.

Reporting for specific materials, systems and methods

We require information from authors about some types of materials, experimental systems and methods used in many studies. Here, indicate whether each material, system or method listed is relevant to your study. If you are not sure if a list item applies to your research, read the appropriate section before selecting a response.

Materials & experimental systems

n/a	Involved in the study	
☐	☒	Antibodies
☒	☐	Eukaryotic cell lines
☒	☐	Palaeontology and archaeology
☒	☐	Animals and other organisms
☐	☒	Clinical data
☒	☐	Dual use research of concern
☒	☐	Plants

Methods

n/a	Involved in the study	
☒	☐	ChIP-seq
☒	☐	Flow cytometry
☒	☐	MRI-based neuroimaging

Antibodies

Antibodies used

MLH1, MSH2, MSH6, PMS2 antibodies were used for MMR-IHC

Validation

For each antibody, proper staining was verified by a pathologist

Clinical data

Policy information about clinical studies

All manuscripts should comply with the ICMJE guidelines for publication of clinical research and a completed CONSORT checklist must be included with all submissions.

Clinical trial registration

NA

Study protocol

NA

Data collection

We collected consecutive prostate cancer patients who were sequenced at Tempus Labs from October 2017 to February 2023 and whose WSIs of prostate biopsies or surgical resections were available. Each case included clinical characteristics, molecular profiles, and digitized WSIs. About 25% of cases have MMR protein IHC. All samples were digitized with either a Philips UFS scanner or an Aperio GT 450 scanner, and 37% of samples were stained at external laboratories. MSI-H/microsatellite-stable (MSS) status was determined using DNA NGS. The NGS assay used in this study, Tempus xT, is a laboratory-developed test used for tumor profiling of solid malignant neoplasms. Samples with equivocal or undetermined MSI status (n=97), samples that failed quality control by pathologists (n=88), and samples with Gleason scores of less than 7 (n=48) were excluded. Reasons for equivocal or undetermined MSI status predominantly result from insufficient tumor purity for the MSI call in NGS, but may also include sequencing of insufficient depth over enough of the assayed microsatellite loci to prevent the prediction from reaching statistical significance, or in rare cases (n=2) multiple tests for the same patient returning conflicting results.
An additional validation set was composed of serial sections of a subset of internal samples stained and scanned at an external site, TruCore Pathology (Little Rock, AR), using an Aperio AT2 scanner.

Outcomes

NA

Plants

Seed stocks

Report on the source of all seed stocks or other plant material used. If applicable, state the seed stock centre and catalogue number. If plant specimens were collected from the field, describe the collection location, date and sampling procedures.

Novel plant genotypes

Describe the methods by which all novel plant genotypes were produced. This includes those generated by transgenic approaches, gene editing, chemical/radiation-based mutagenesis and hybridization. For transgenic lines, describe the transformation method, the number of independent lines analyzed and the generation upon which experiments were performed. For gene-edited lines, describe the editor used, the endogenous sequence targeted for editing, the targeting guide RNA sequence (if applicable) and how the editor was applied.

Authentication

Describe any authentication procedures for each seed stock used or novel genotype generated. Describe any experiments used to assess the effect of a mutation and, where applicable, how potential secondary effects (e.g. second site T-DNA insertions, mosiacism, off-target gene editing) were examined.
