# Supplementary material for: Development and validation of a deep learning-based microsatellite instability predictor from prostate cancer whole-slide images
Source: NPJ Precis Oncol. 2024 Apr 9;8:88. doi: 10.1038/s41698-024-00560-7 (PMC11004110; doi:10.1038/s41698-024-00560-7)
Supplement: Supplementary file 2 — Supplementary Information [file 41698_2024_560_MOESM2_ESM.docx]

# Supplementary Information

## Hyperparameter tuning

Five-fold cross-validation within the training set was used to perform hyperparameter tuning to select for learning rate, weight decay, dropout rate, patience and minimum delta for early stopping, input image magnification, and color augmentation parameters. Learning rate, weight decay, and dropout rate were combined into one grid search, while other hyperparameters were swept individually on a grid search. The ranges of the search were image magnification: 5x, 10x, 20x; learning rate: 1e-7~1e-4; weight decay: 1e-5~1e-2; dropout rate: 0.1~0.5; early stopping patience: 5~20; early stopping minimum delta: 0~0.001; color augmentation: spans the entire range of color jittering. The set of hyperparameters that achieved the highest ensemble validation AUC during cross-validation was selected as optimal, which was image magnification 20x, learning rate 1e-6, weight decay 1e-4, dropout rate 0.1, early stopping patience 10, early stopping minimum delta 0.00025, slide-level brightness augmentation 0.25, slide-level contrast augmentation 0.5, slide-level saturation augmentation 0.25, and slide-level hue augmentation 0.04.

## Model generalizability

Our unique paired serial section validation set allowed us to measure the impact of methods on model robustness, as measured by the correlation between predictions between the internally and externally stained and scanned serial sections, in addition to overall model predictive performance. Our model showed a correlation of 0.73 (95% CI [0.60, 0.83]) (Supplementary Fig. 1).

We conducted exploratory analyses to assess the impact of several methods on our MSI-H predictor. The following methods resulted in notable effects (Supplementary Fig. 6). First, compared with tile-level color augmentation, slide-level color augmentation showed improvements in model generalizability, with a significant increase in R of 0.13 (95% CI [0.05, 0.26]) between predictions on the internally and externally stained and scanned slides in the paired validation set. On the other hand, contrary to what some other studies have suggested, self-supervised pre-training reduced model performance and generalizability, with a decrease in AUC of -0.02 (95% CI [-0.06, 0.01]) and -0.09 (95% CI [-0.19, -0.00]) on the internally and externally stained and scanned slides, respectively, and a significant change in R of -0.35 (95% CI [-0.47, -0.18]). The 95% CIs of the difference in metrics were calculated by bootstrapping the paired prediction scores from two models with 1000 bootstrap samples.

## Lynch Syndrome Considerations in Study Cohort

In our study, for estimating the prevalence of Lynch syndrome in MSI-H prostate tumors, it is pertinent to consider the diagnostic capabilities of the Tempus xT assay. This assay, primarily designed for somatic mutation detection, also can detect potential germline genes related to Lynch syndrome when normal matched tissue is also sequenced.[^1^](https://paperpile.com/c/E5HlWl/jAYx) This potential germline variant detection aligns with American College of Medical Genetics (ACMG) guidelines, which recommends reporting certain cancer-related incidental findings identified during genetic testing.[^2^](https://paperpile.com/c/E5HlWl/bIoj) Despite its capacity for potential germline variant detection, xT is not a stand-alone germline test and has not been validated for assessment of hereditary disease risk. For example, the assay has limited capacity to detect CNV germline mutations and cannot detect large deletions/duplications or gene rearrangements (EPCAM deletions [^3^](https://paperpile.com/c/E5HlWl/bcRn) and recurrent MSH2 inversions of exons 1-7 [^4^](https://paperpile.com/c/E5HlWl/rTPZ)). As a result, while the xT panel can identify potential germline variants, the available data cannot be used to definitively rule out Lynch syndrome and is likely an underestimation of Lynch syndrome prevalence in this patient population. Acknowledging these limitations, Lynch syndrome was detected in our study cohort at a prevalence of 0.41% in cases with matched tumor-normal samples (n=20/4888 [Lynch syndrome positive/xT prostate cancer patients]).

## Morphological analysis

A U.S. board-certified anatomic pathologist provided their blinded assessments of 60 slides (15 in each of the true positive, true negative, false positive, false negative categories) on lymphocytes within and around the tumor, predominant growth pattern, and histology of the highest Gleason pattern on each slide. Several examples from each category are shown in Supplementary Fig 2(b), and findings from the pathologist’s morphological assessment are summarized in Supplementary Fig. 2(c).

As expected, lymphocytic infiltrate is higher in MSI-H than in MSS cases. In terms of correlation with model predictions, lymphocytic infiltrate is higher in the FP category than the TN category but is not substantially differentiated between the TP and FN groups. The model was able to identify MSI-H with several predominant growth patterns and histology patterns across a range of Gleason scores.

Acknowledging this is a small dataset, we noted a few additional observations: Cases with high Gleason scores and “solid” histological growth patterns were frequently classified as MSI-H by the model, resulting in both TPs and FPs. Among these FPs, tiles consisting mostly of blood seemed to be a recurring theme among high-attention tiles. Interestingly, all cases with small glandular proliferation (Gleason pattern 3) predominant growth pattern (n=5) were correctly predicted as MSS.

###### Supplementary Table 1. Patient clinical and molecular features

**
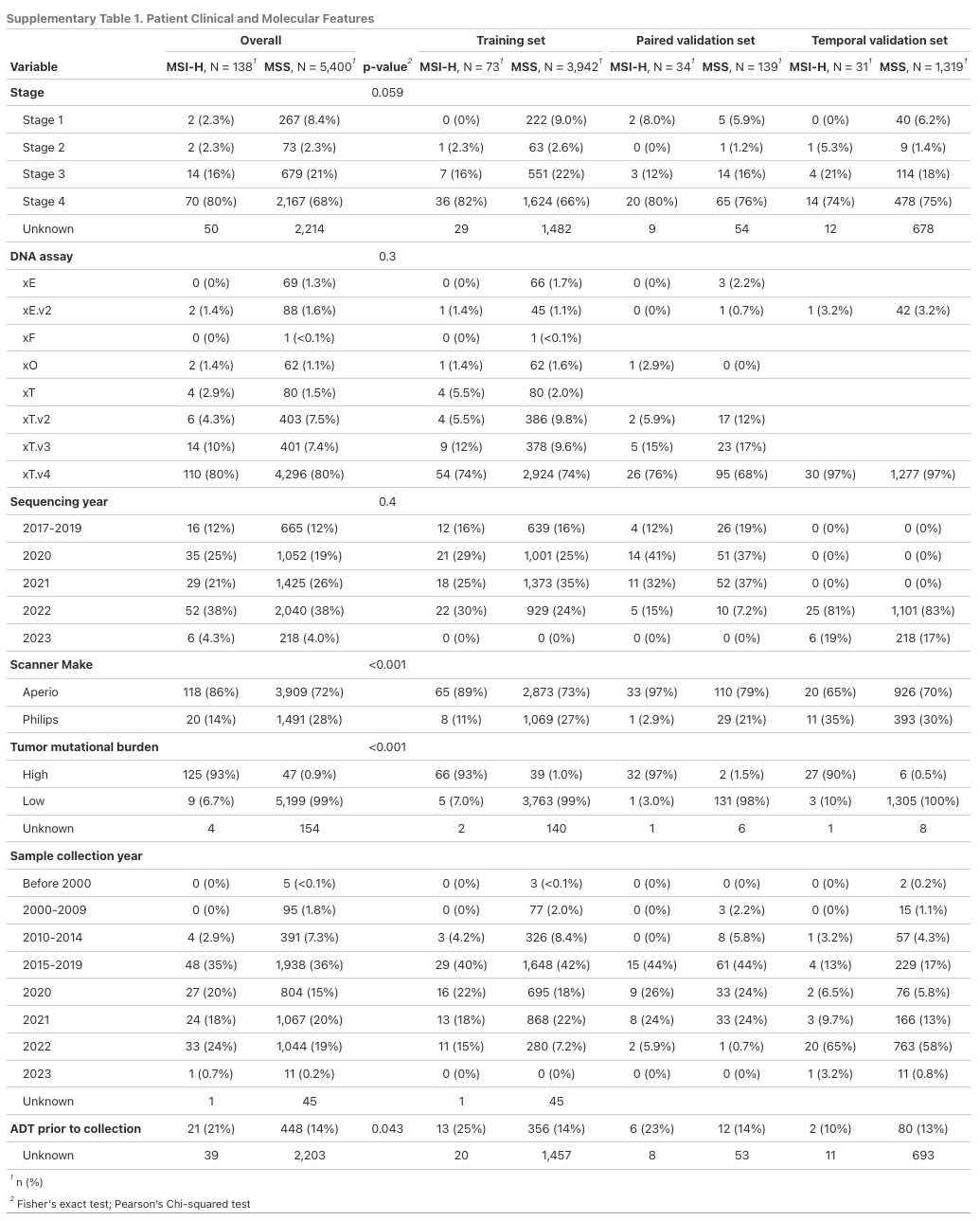
**

######

###### Supplementary Table 2. Multivariate logistic regression model for clinical and molecular variables in patient cohort.

######
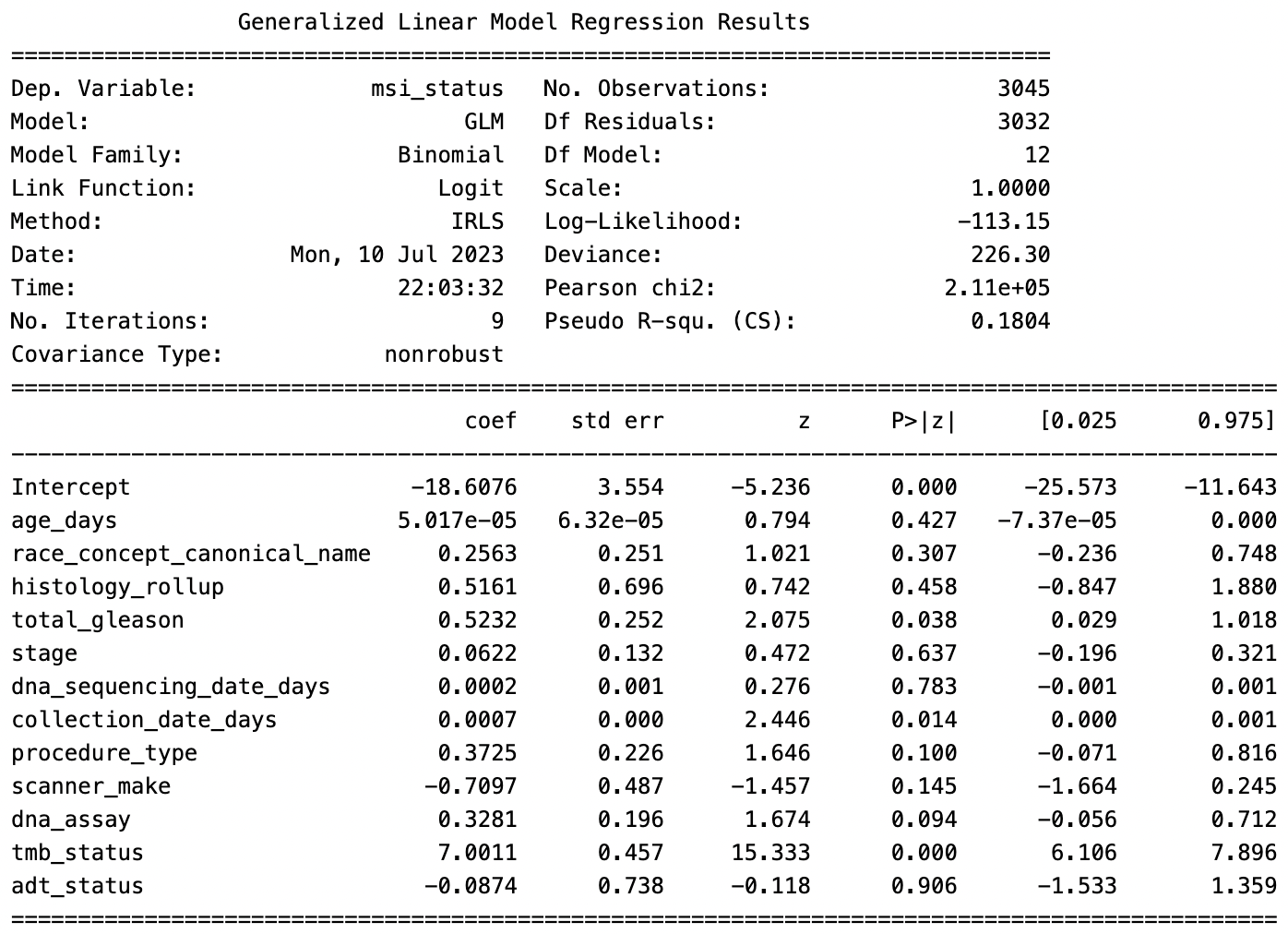


**
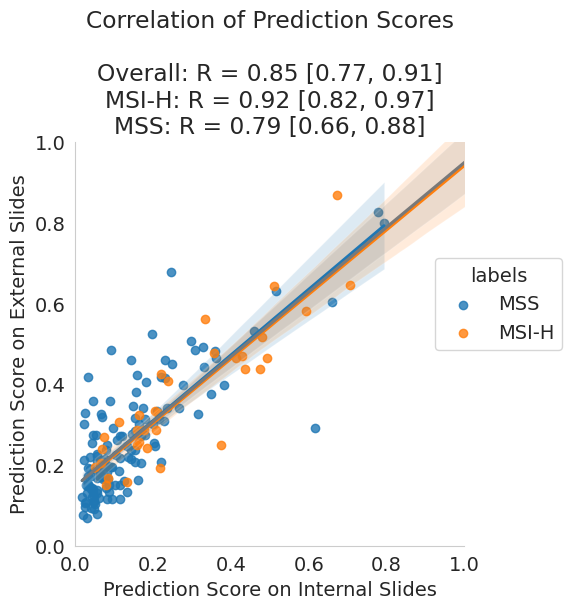
**

|  | **R [95% CI]** |
| --- | --- |
| Philips UFS (internal) vs. Aperio AT2 (external), n=30 | 0.70 [0.27, 0.88] |
| Aperio GT450 (internal) vs. Aperio AT2 (external), n=143 | 0.90 [0.83, 0.94] |

###### Supplementary Figure 1. Correlation between the prediction scores of the internally and externally stained and scanned slides in the paired validation set. (a) The figure contains the Pearson correlation coefficients and the fitted regression lines for all cases (grey) and MSI-H (orange) and MSS (blue) subsets, respectively. The shared areas represent the 95% confidence intervals of the regression estimates. (b) Subgroup analysis by scanner type of the internal scans.


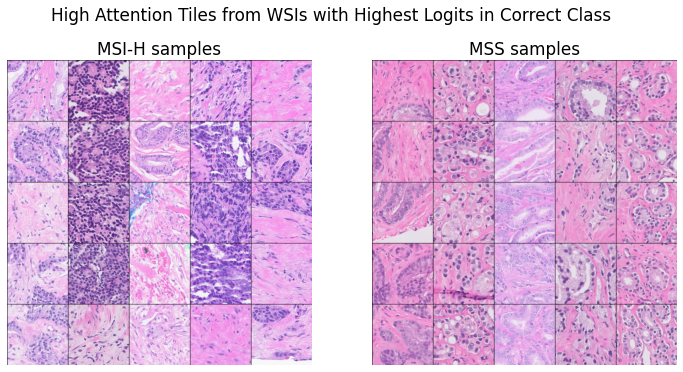


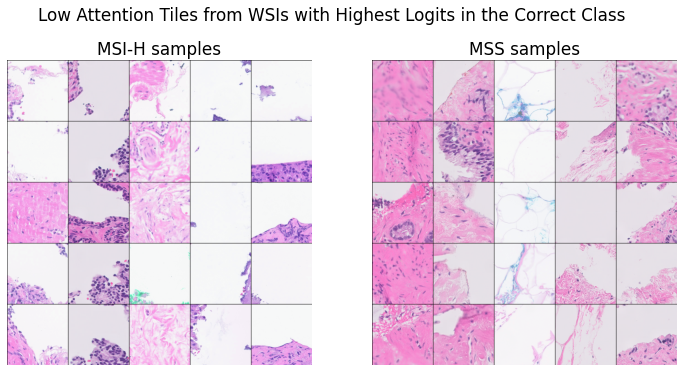


**True Positive Samples**


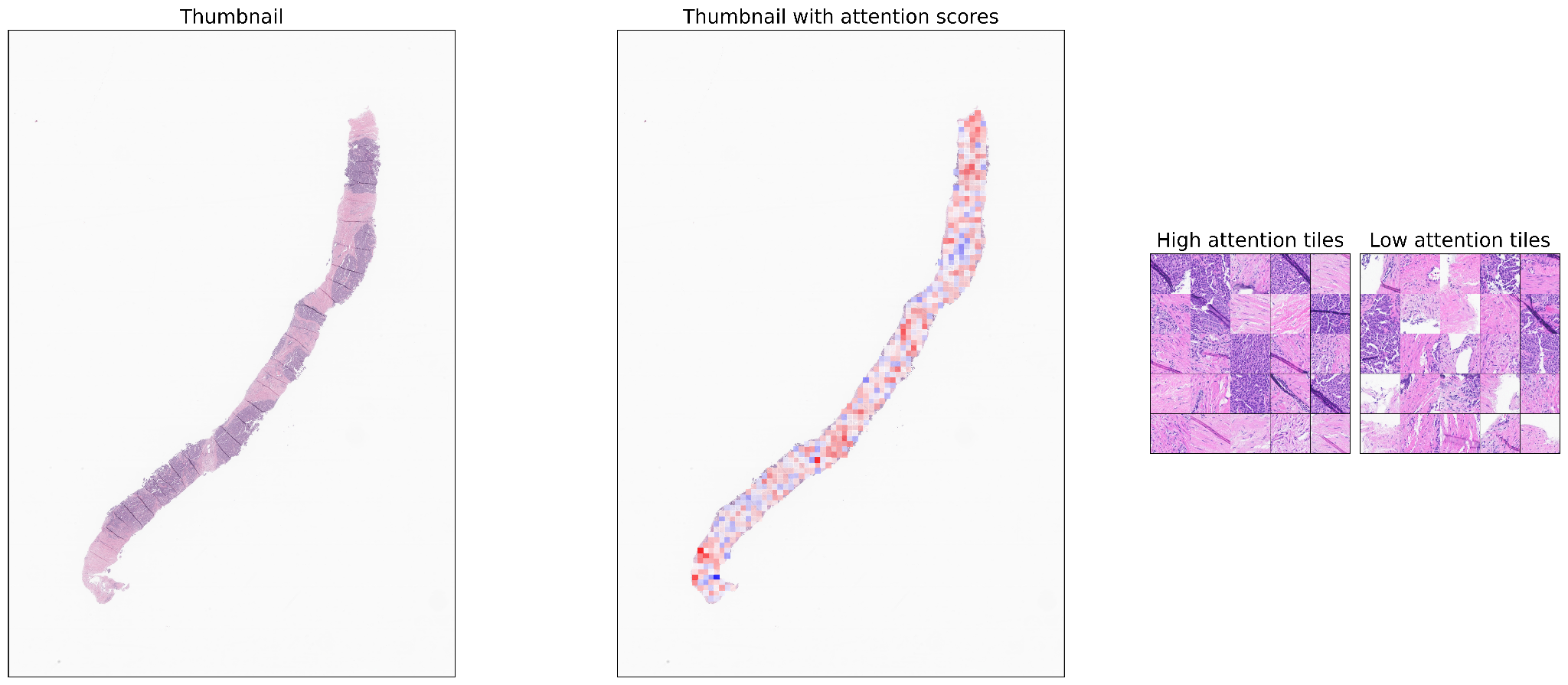


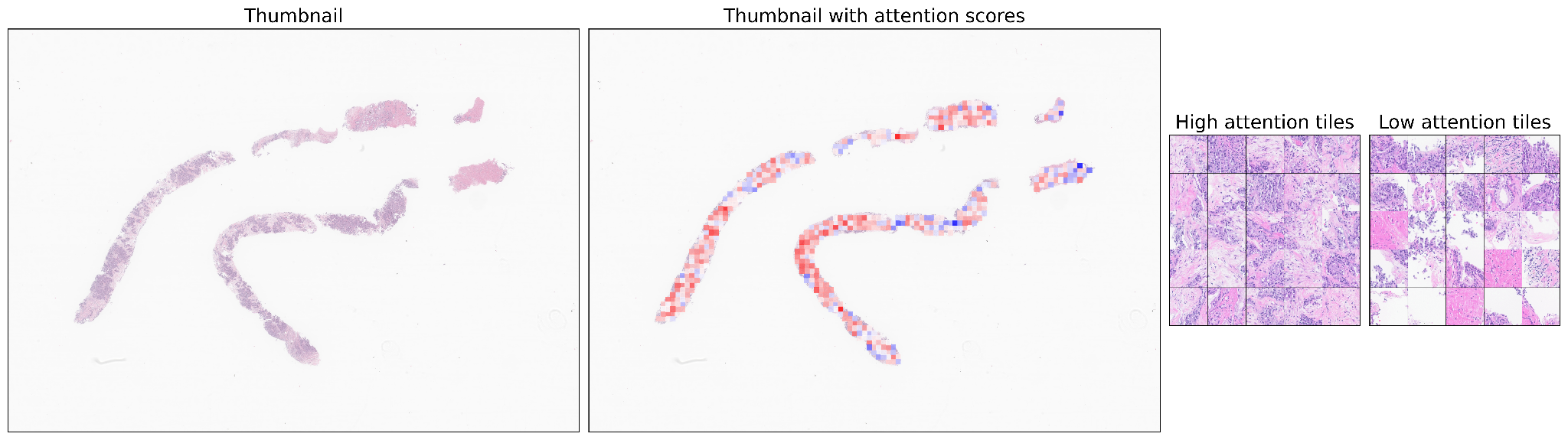


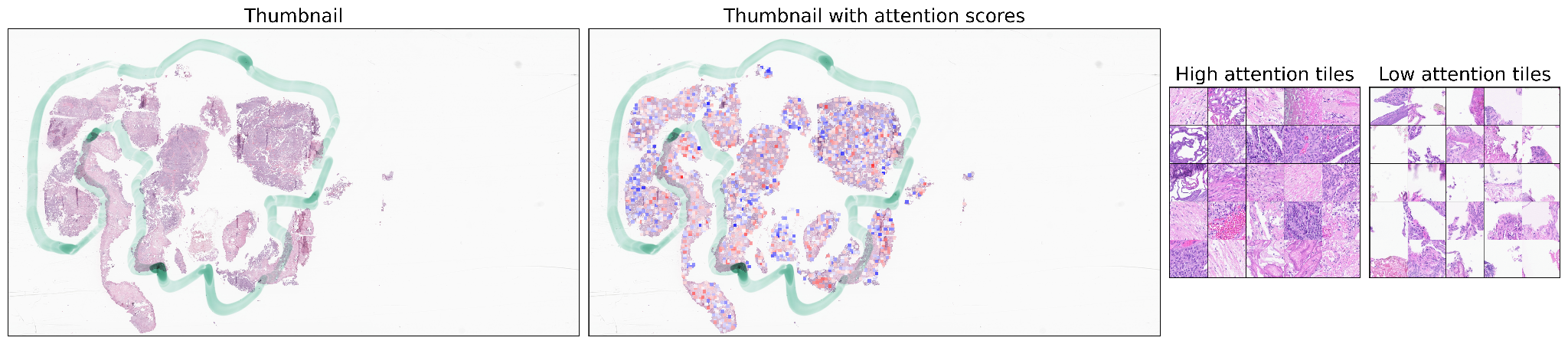


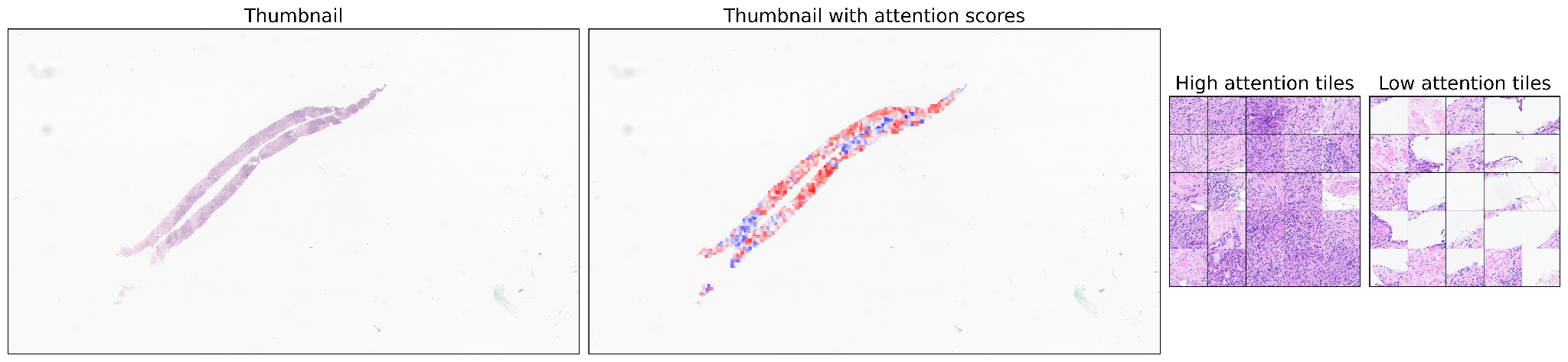


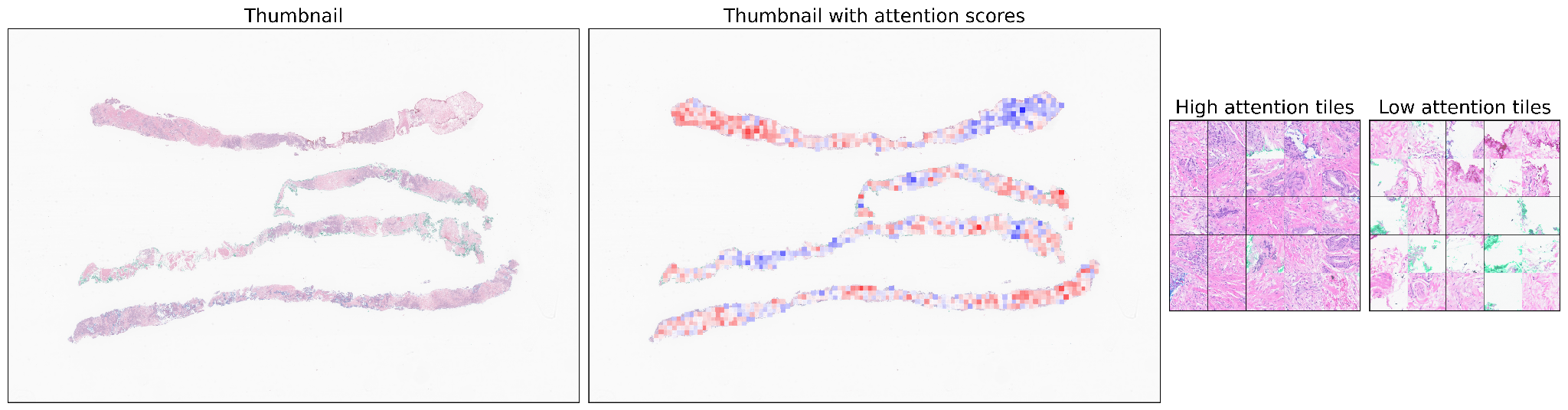


**False Negative Samples**


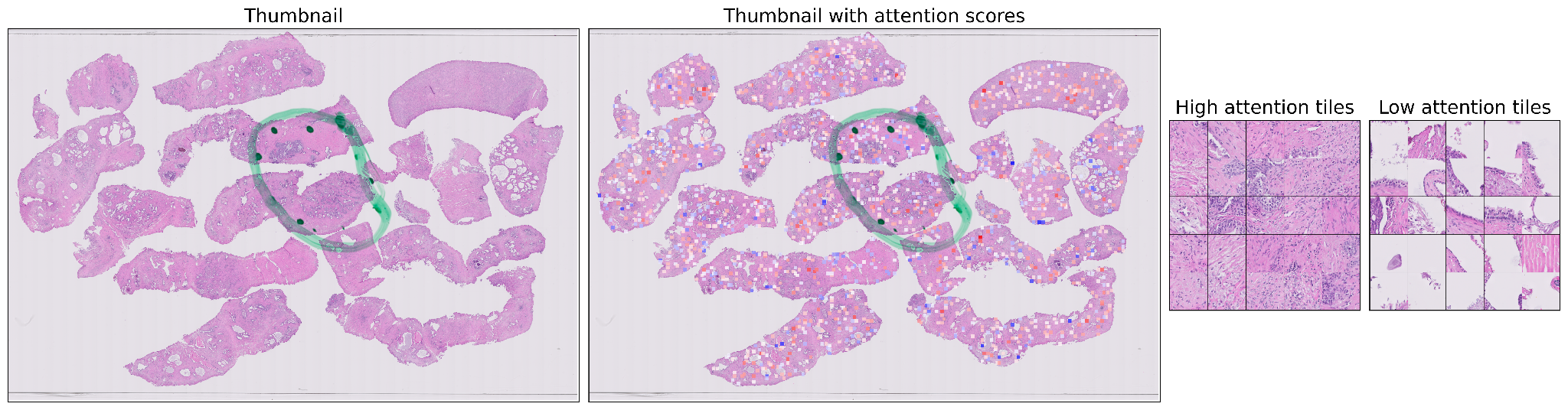


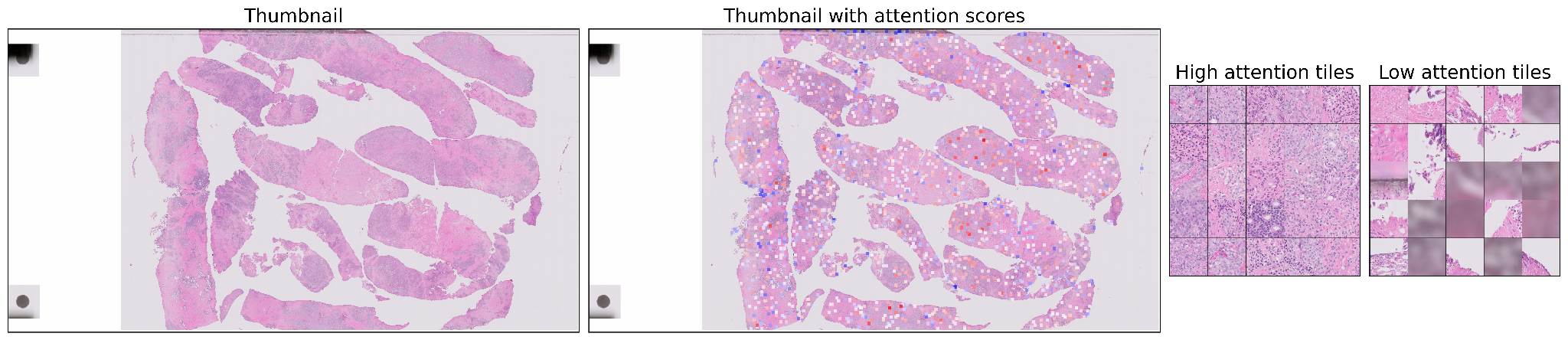


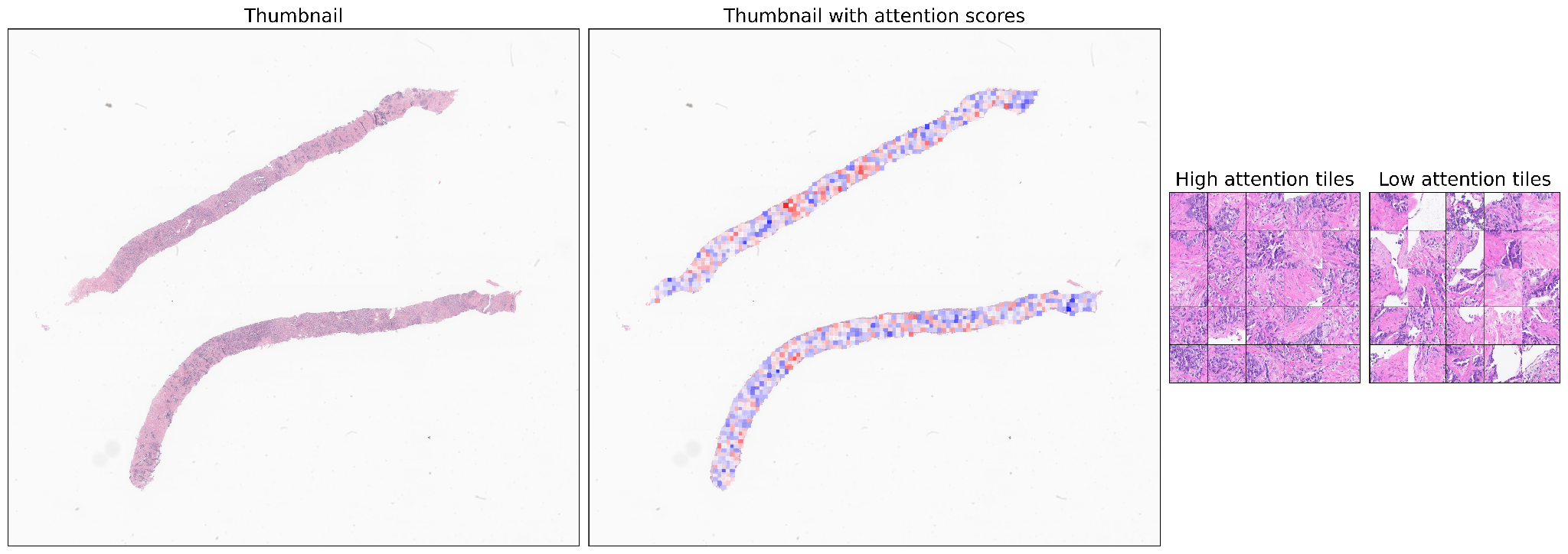


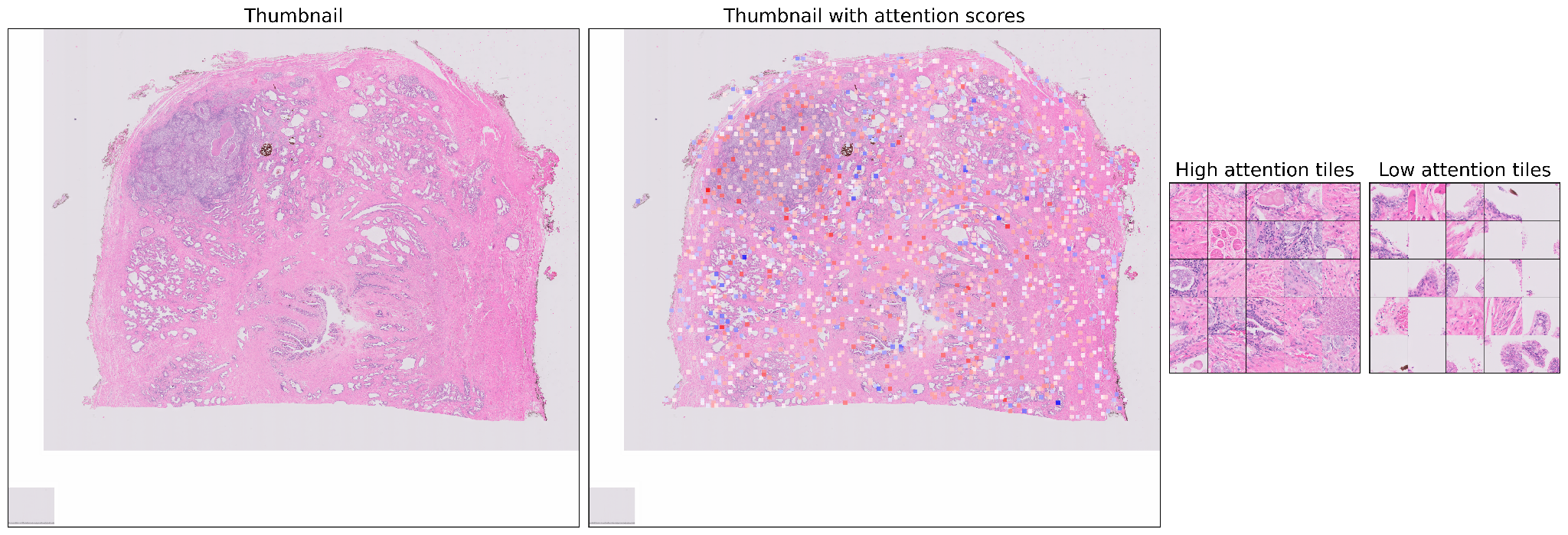


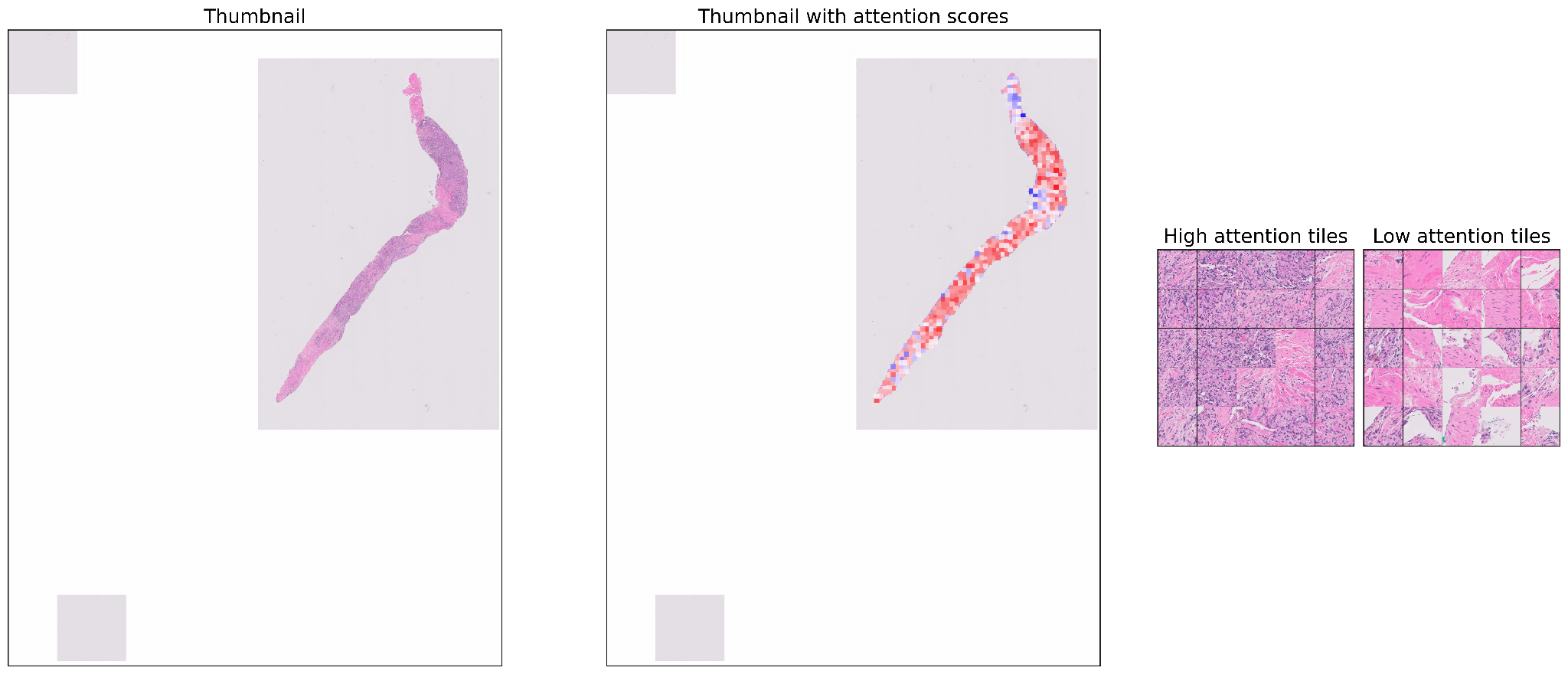


**True Negative Samples**


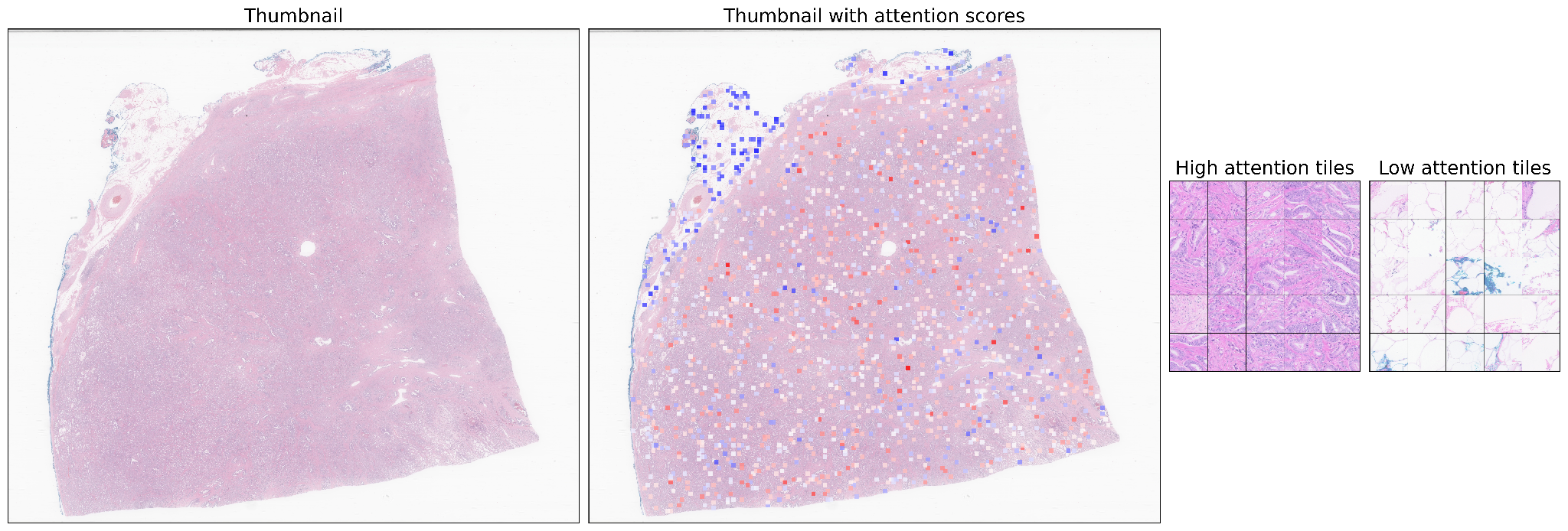


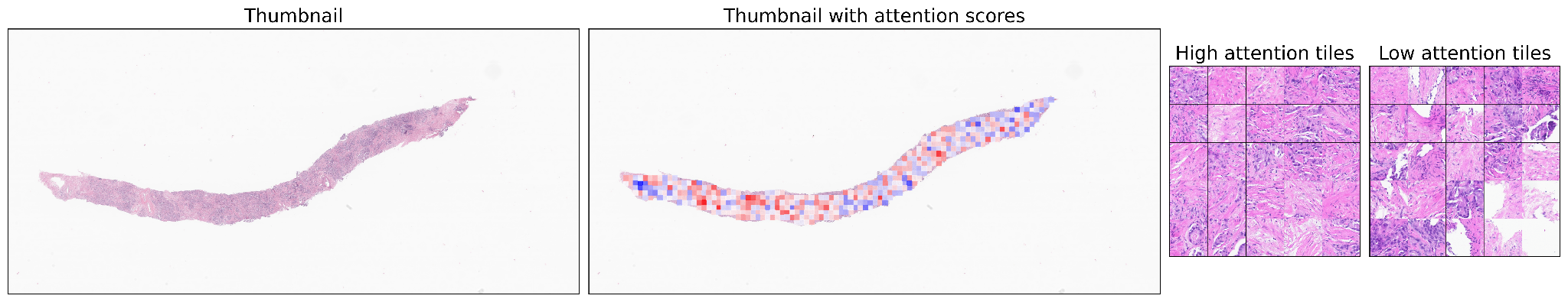


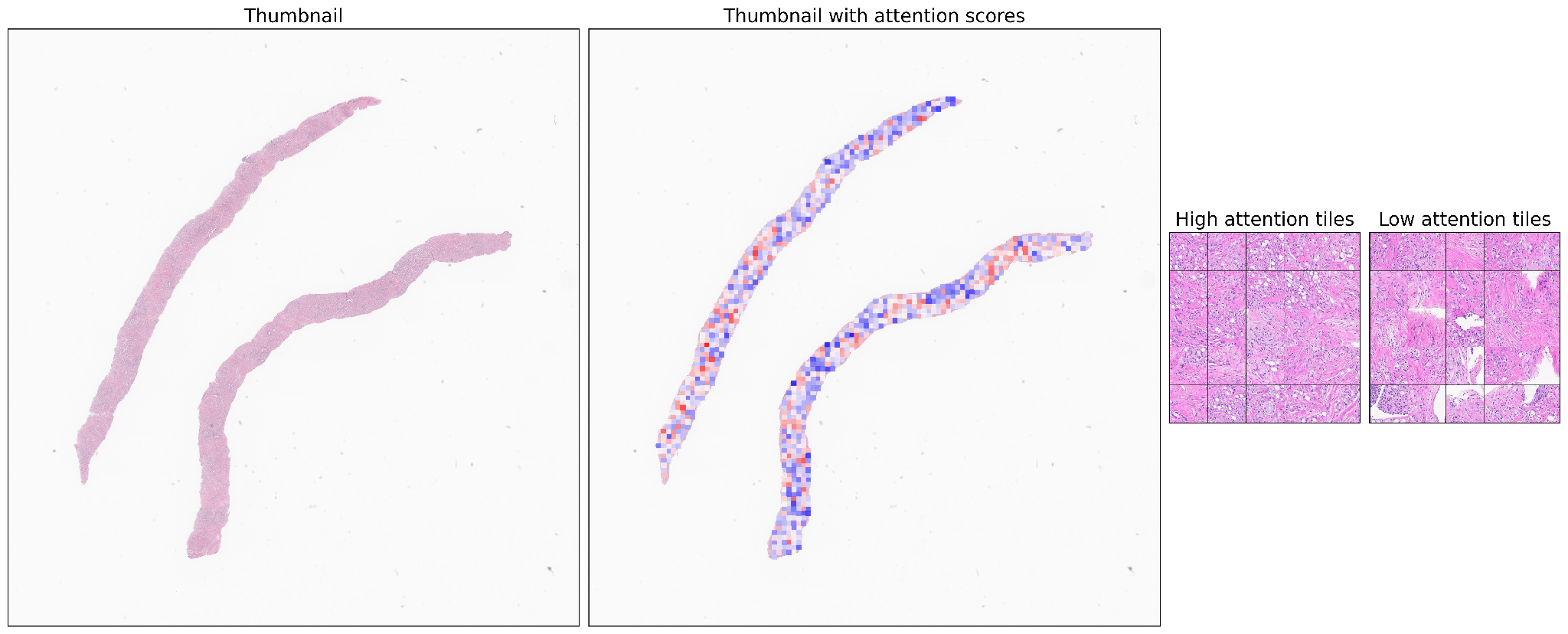


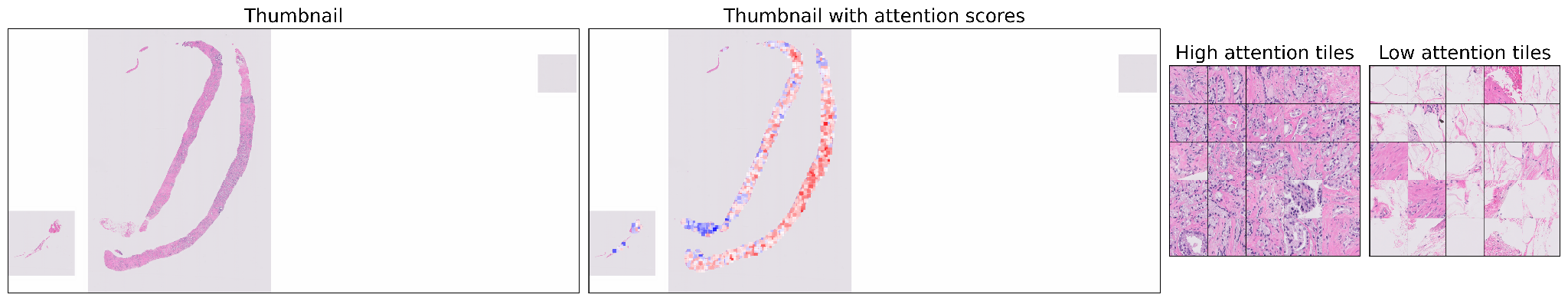


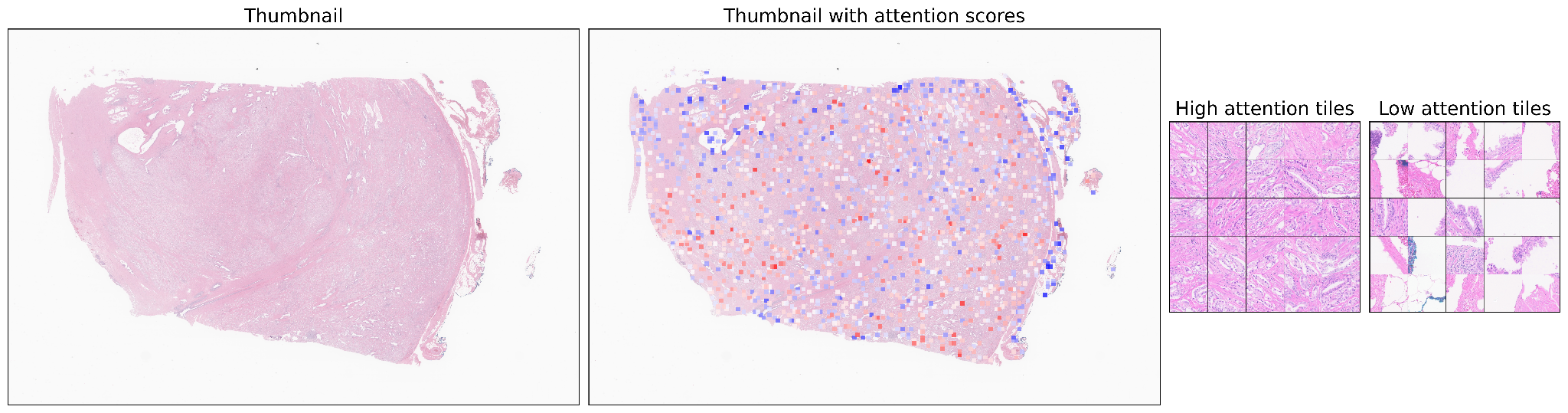


**False Positive Samples**


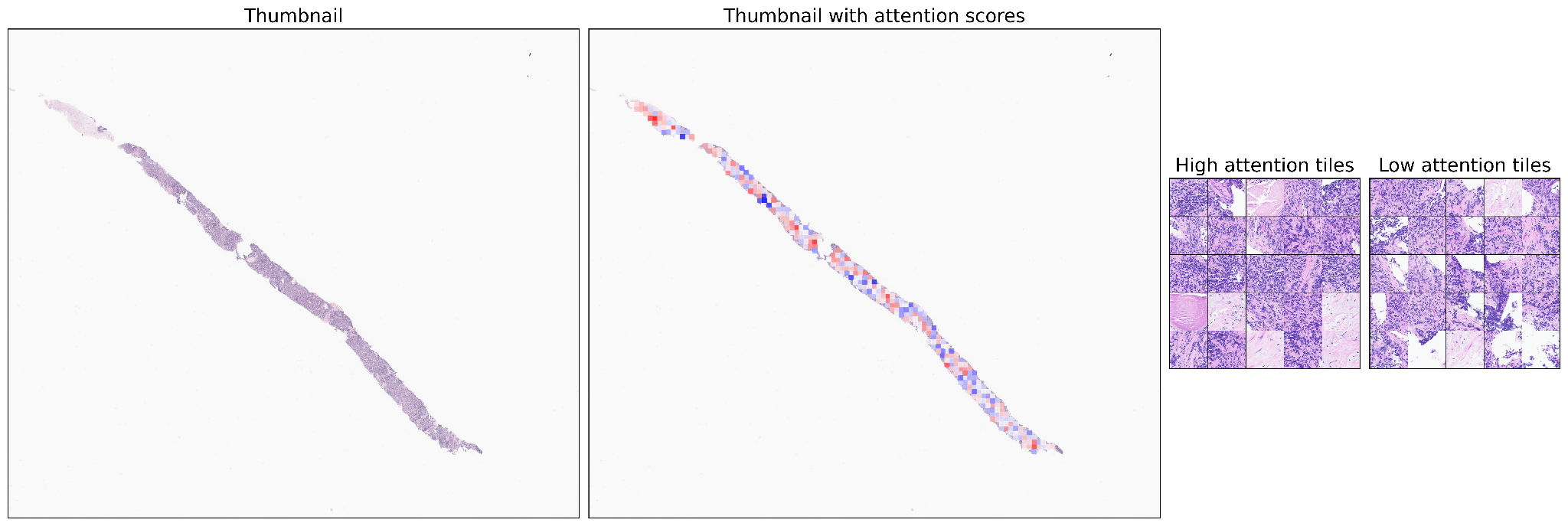


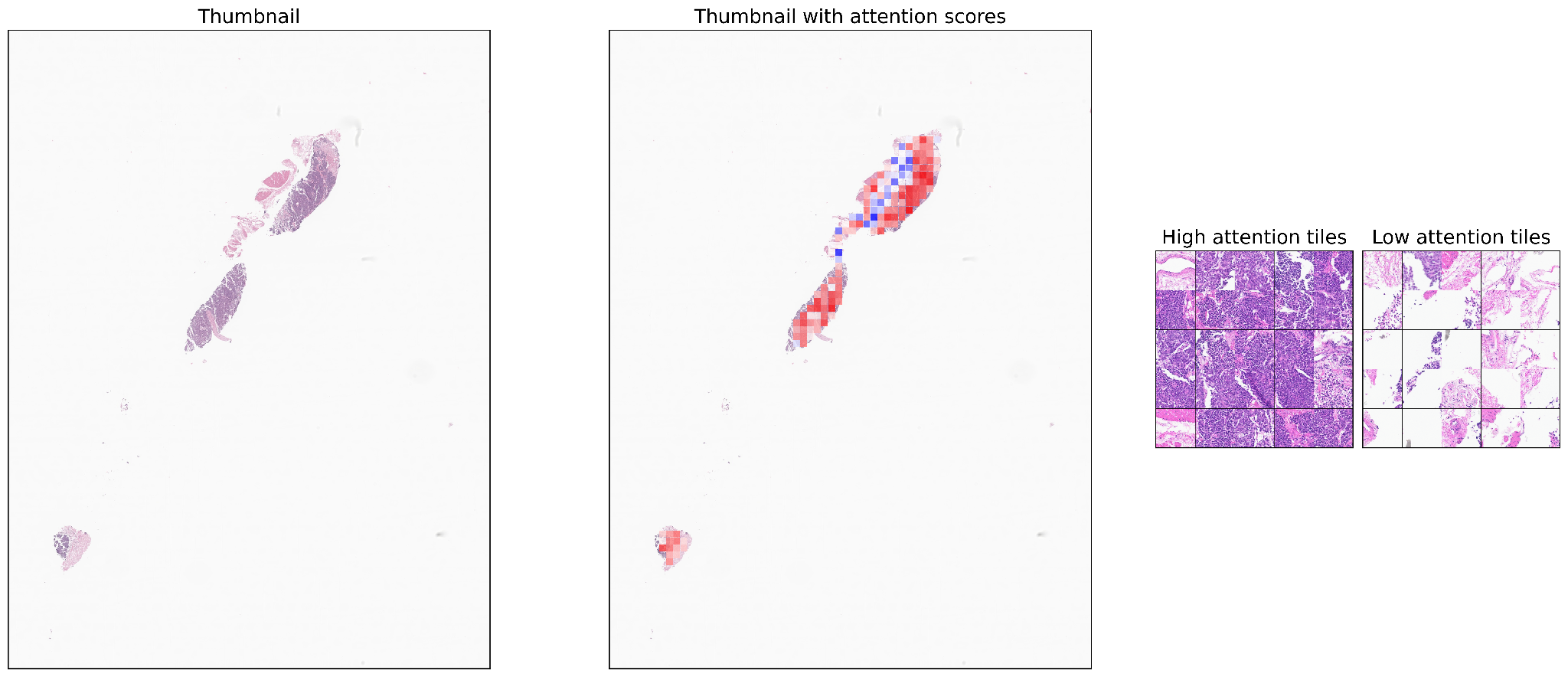


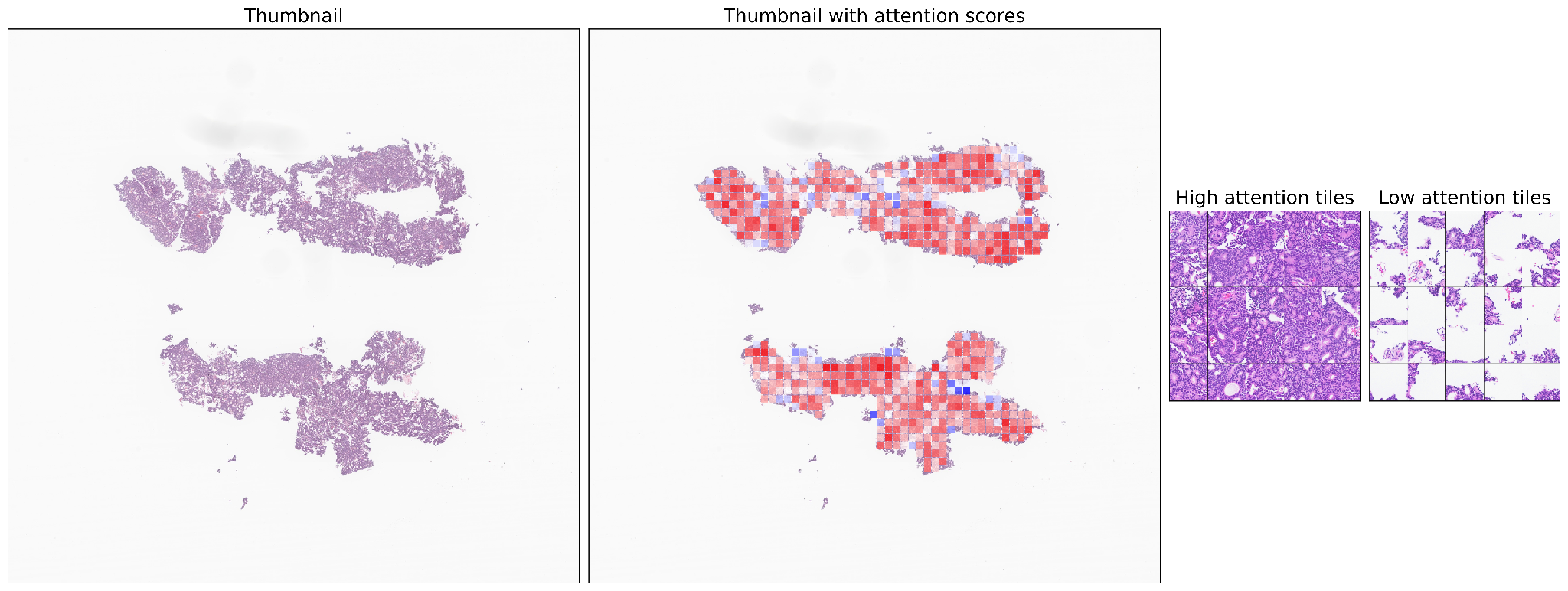


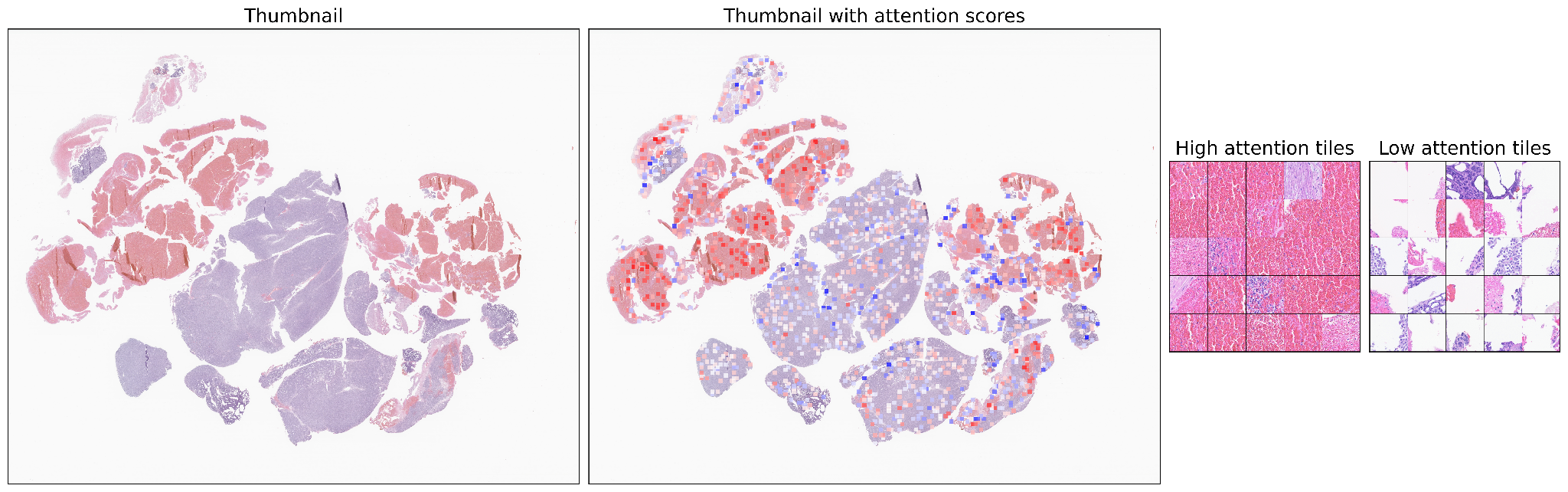


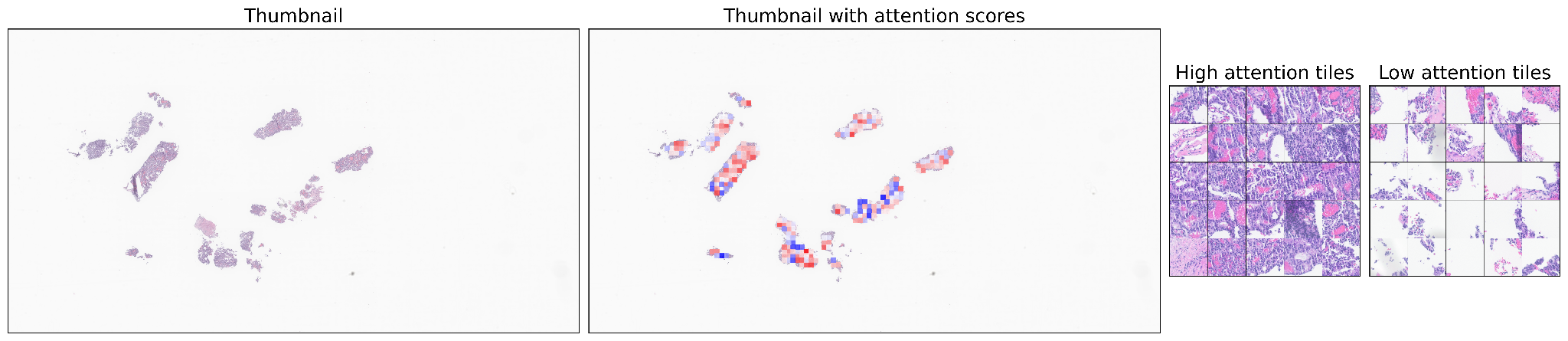


|  |  | **MSI-H** | | **MSS** | |
| --- | --- | --- | --- | --- | --- |
|  |  | **TP** | **FN** | **TN** | **FP** |
| **lymphocytes within/around tumor** | **low** | 9 | 9 | 14 | 12 |
|  | **medium** | 4 | 2 | 1 | 3 |
|  | **high** | 2 | 4 | 0 | 0 |
| **predominant growth patterns** | **cribriform** | 11 | 8 | 8 | 5 |
|  | **solid** | 4 | 0 | 0 | 10 |
|  | **cords/chains** | 0 | 0 | 1 | 0 |
|  | **single separate glands** | 0 | 0 | 5 | 0 |
|  | **hypernephroid pattern** | 0 | 1 | 1 | 0 |
| **total Gleason score** | **7** | 0 | 2 | 8 | 0 |
|  | **8** | 8 | 4 | 4 | 4 |
|  | **9** | 5 | 5 | 3 | 2 |
|  | **10** | 2 | 4 | 0 | 9 |
| **histology of highest Gleason pattern** | **cribriform** | 8 | 5 | 12 | 4 |
|  | **solid** | 5 | 8 | 0 | 10 |
|  | **comedonecrosis** | 2 | 1 | 1 | 1 |
|  | **single cell** | 0 | 0 | 2 | 0 |
|  | **hypernephroid pattern** | 0 | 1 | 0 | 0 |

###### Supplementary Figure 2. (a) High-attention tiles (top) and low-attention tiles (bottom) from WSIs that received the highest prediction scores in their corresponding correct class. (b) Examples of true positive, false negative, true negative, and false positive slides showing the thumbnail of each WSI, attention score heatmaps overlaid on the WSI (red corresponds to high attention scores and blue corresponds to low attention scores), and top high/low-attention tiles from each slide. (c) Results from blinded pathologist assessment of representative slides in each category of model prediction.

1. Paired validation set (internal scans)


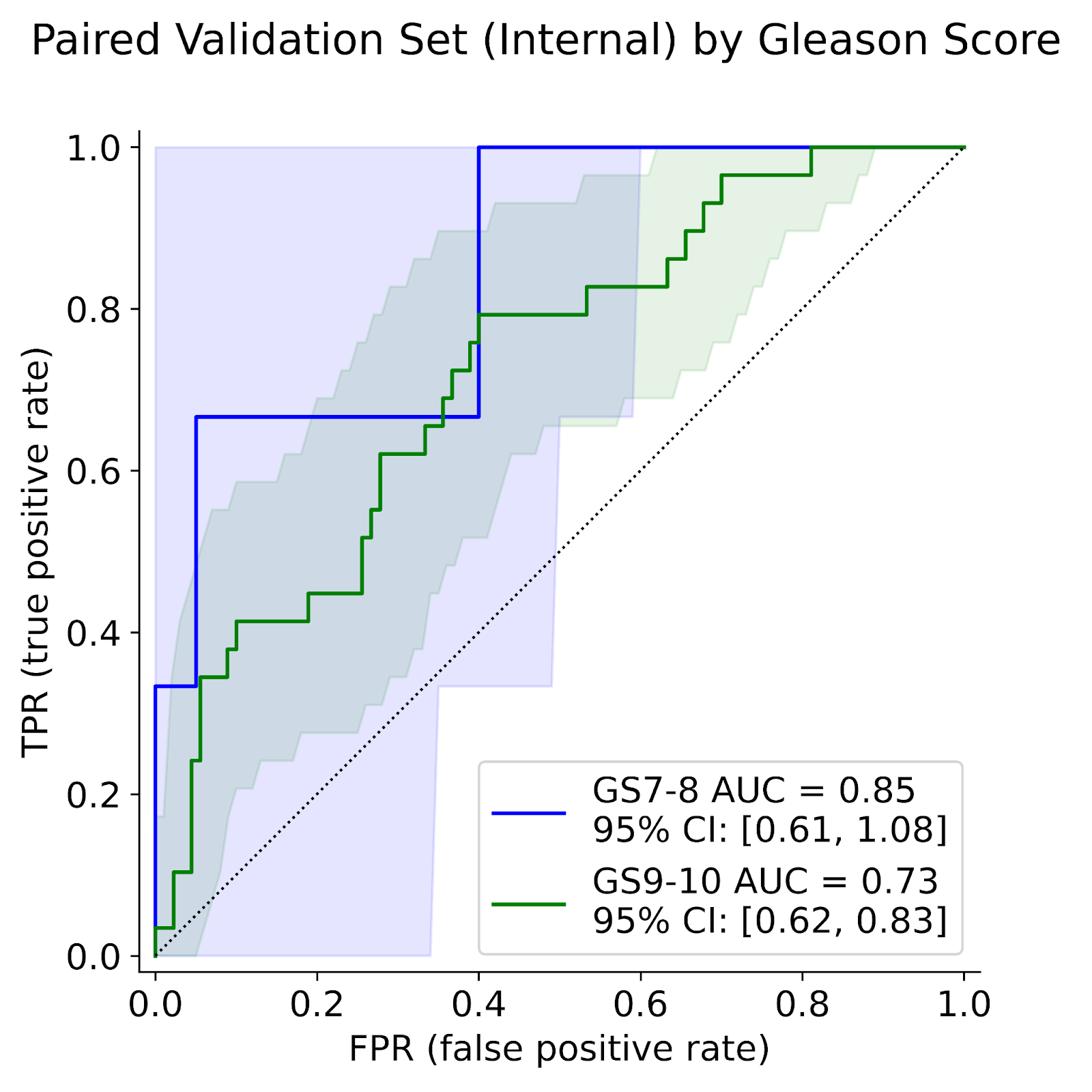

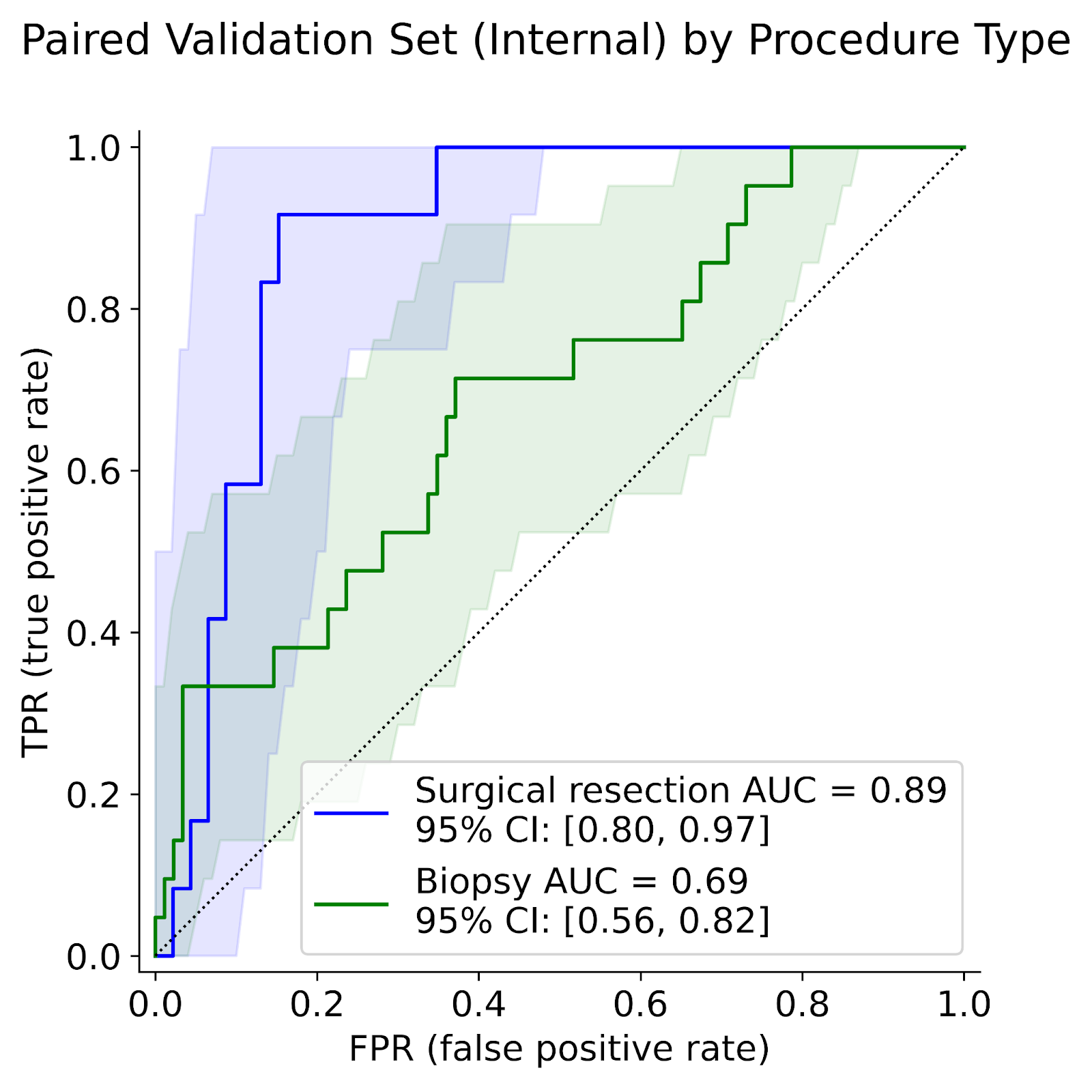


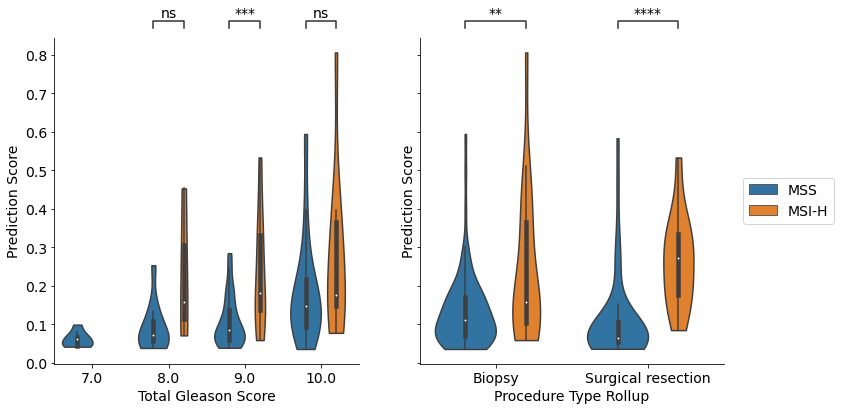


1. Paired validation set (external scans)


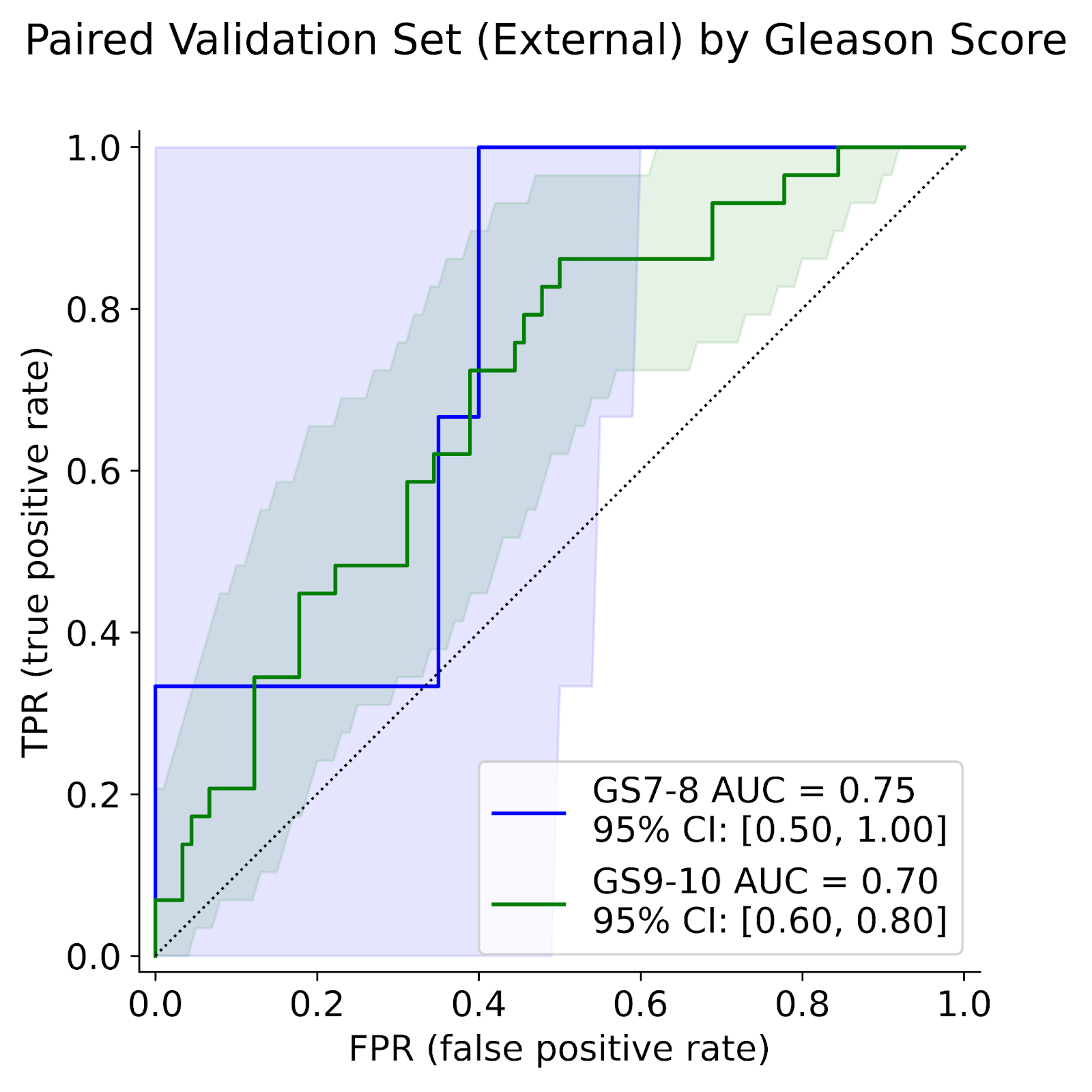

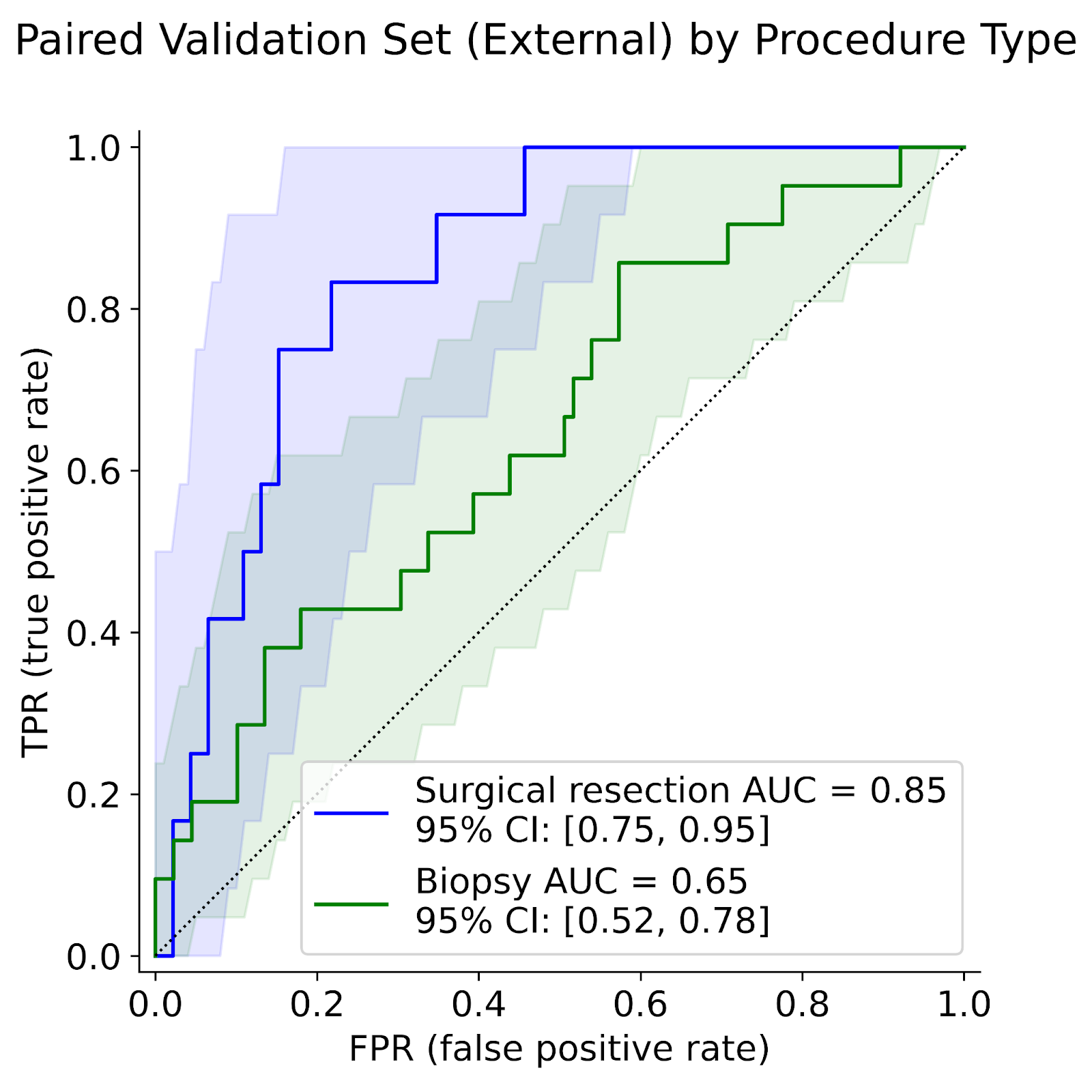


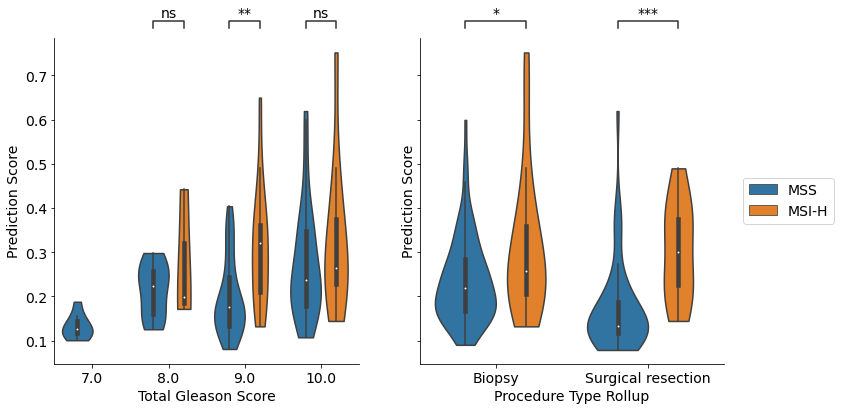


######

1. Temporal validation set


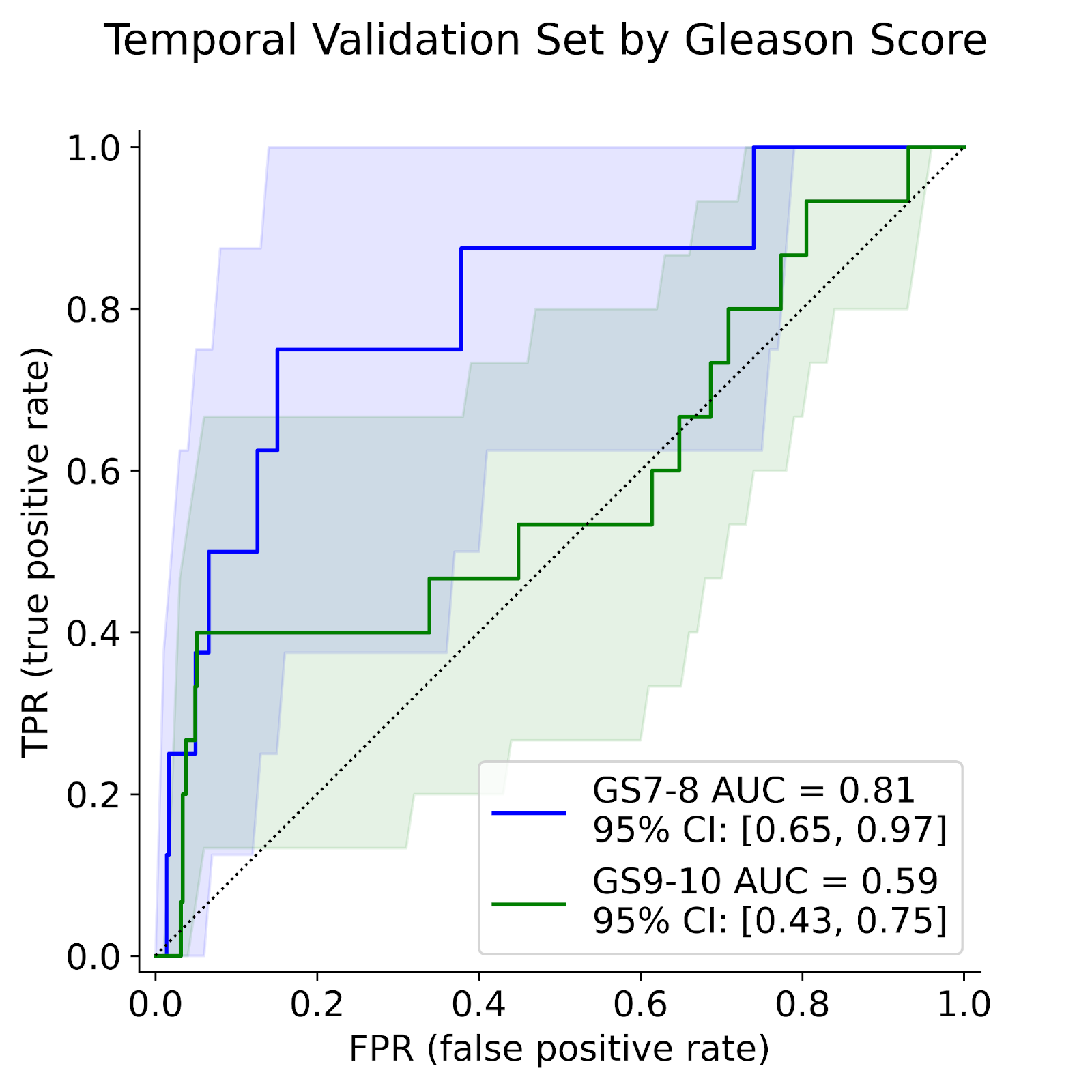

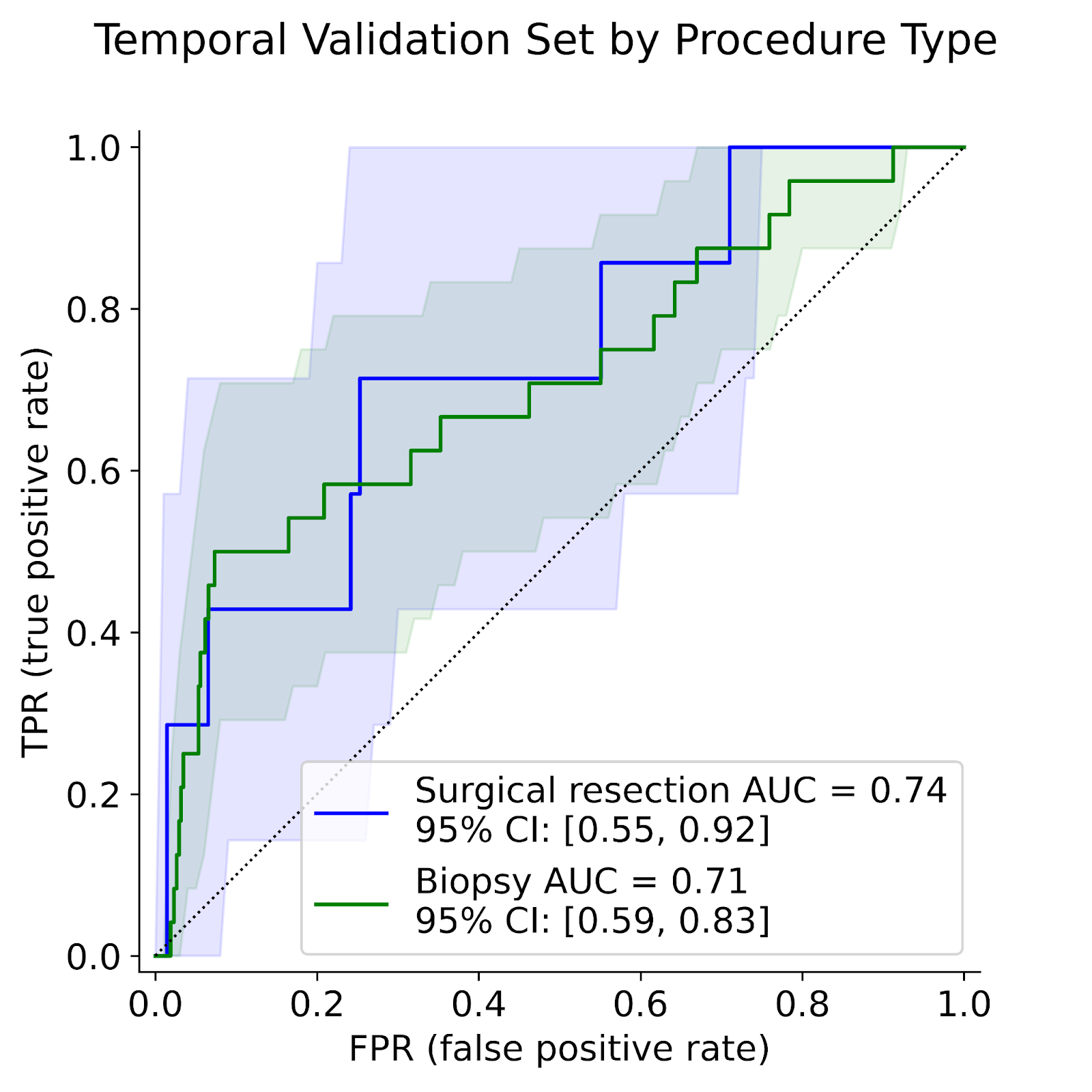


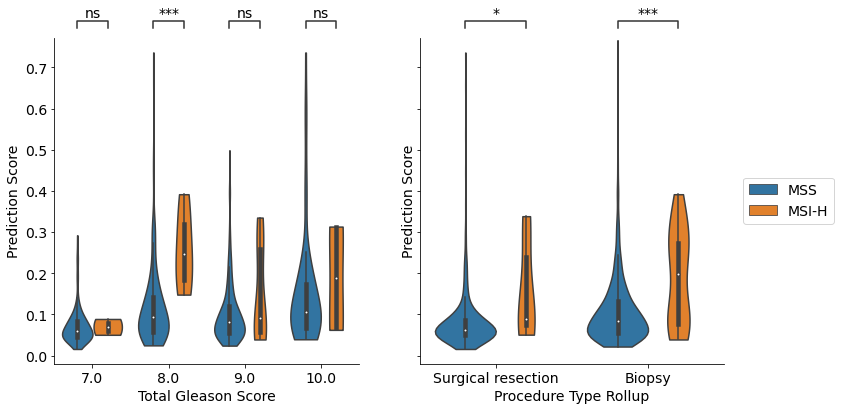


###### Supplementary Figure 3. Receiver operating characteristic (ROC) curves and violin plots of prediction scores for various clinical subgroups in (a) internal scans in the paired validation set, (b) external scans in the paired validation set, and (c) temporal validation set. The shared areas represent the 95% confidence intervals of the ROC curves. P-value annotation legend: ns: p ≤ 1, *: 0.01 < p ≤ 0.05, **: 0.001 < p ≤ 0.01, ***: 0.0001 < p ≤ 0.001, ****: p ≤ 0.0001.


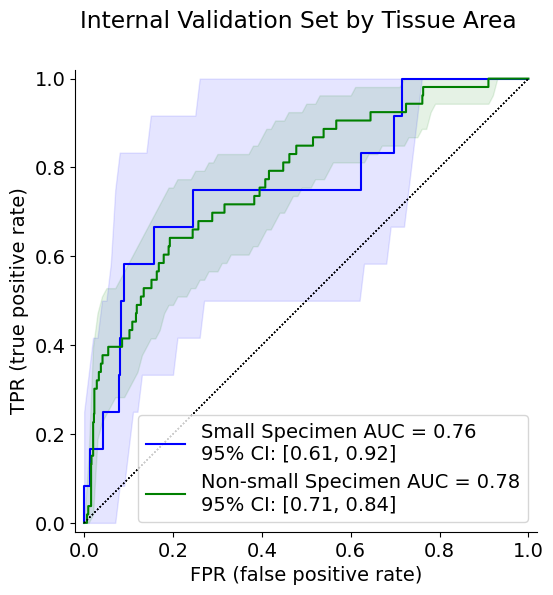

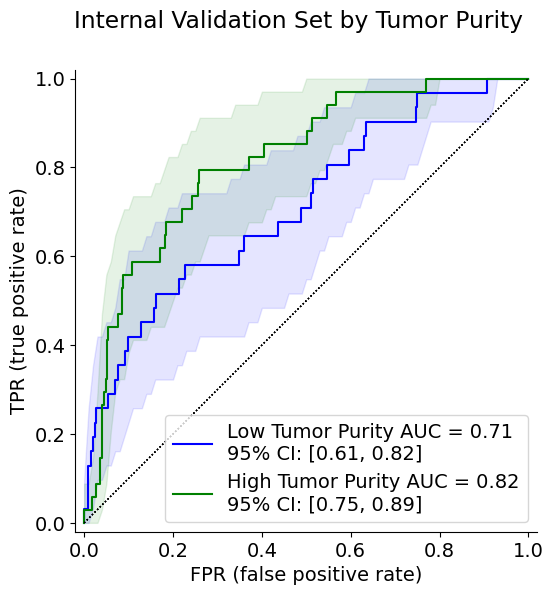


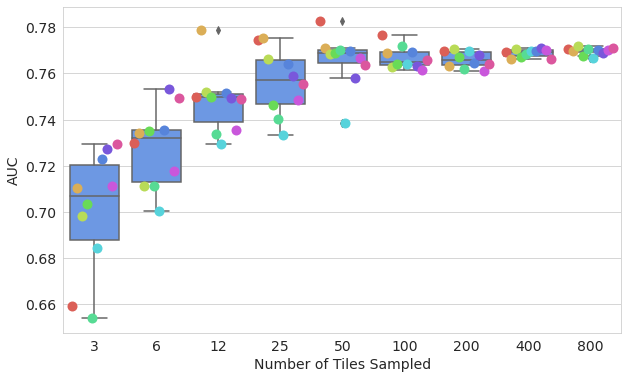

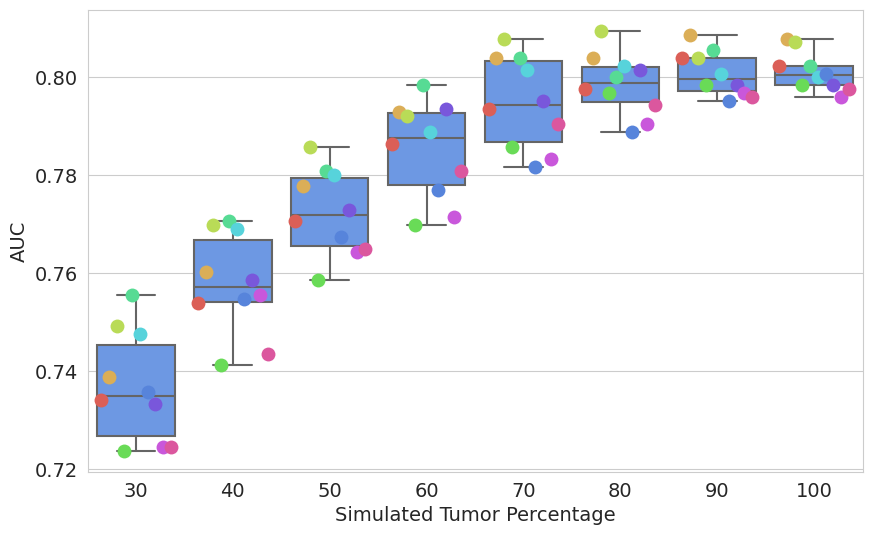


###### Supplementary Figure 4. (a) Receiver operating characteristic (ROC) curves for tissue area and tumor purity subgroups in the internal scans in the pooled validation set that combines the internal scans of the paired validation set and the temporal validation set. The threshold value for both subgroups is the first quartile within the validation set, which is 9.35 mm^2^ for tissue area and 50% for tumor purity. The shared areas represent the 95% confidence intervals of the ROC curves. (b) Effect of tissue area and tumor purity on model performance from the simulation experiment on the combined internal test set. Data points within each box plot demonstrate variations when 10 random different seeds were used for tile sampling. Elements of box plot: center line, median; box limits, upper and lower quartiles; whiskers, 1.5x interquartile range; points, outliers.


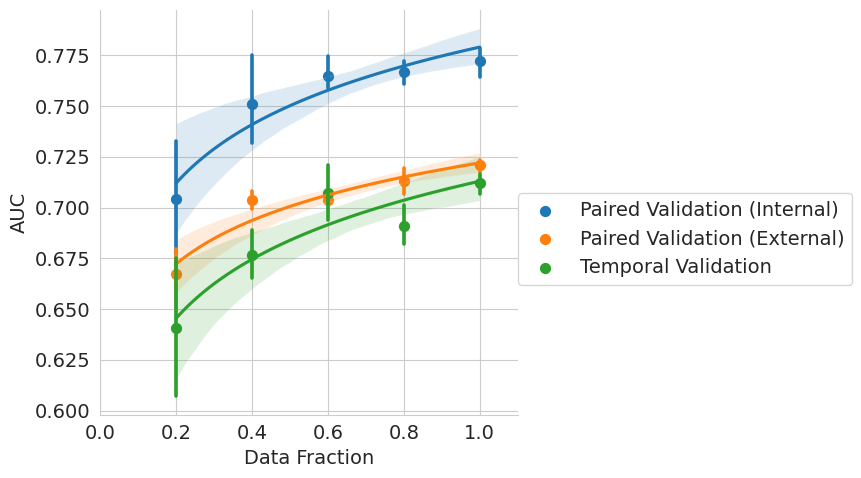


###### Supplementary Figure 5. Area under the receiver operating characteristic curves (AUC) trends on the three validation sets when various fractions of the training data were used. The lines are fitted linear regressions with the explanatory variable on the log scale. The shared areas represent the 95% confidence intervals of the regression estimates. Points and error bars represent the mean and the 95% confidence interval at each data fraction.


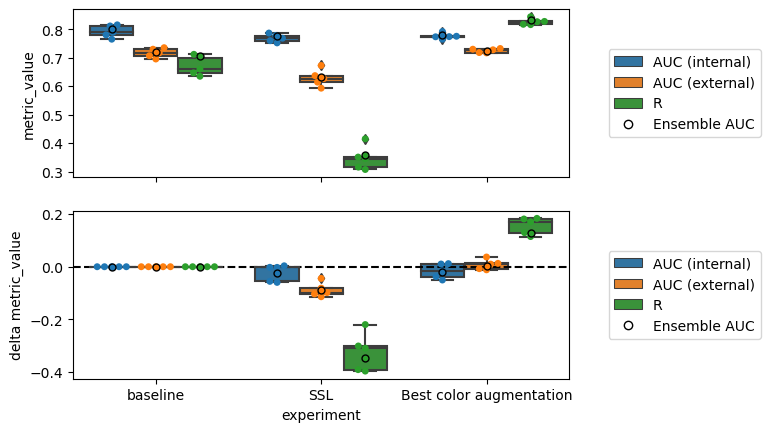


###### Supplementary Figure 6. The difference in metrics caused by self-supervised learning pretraining and strong slide-level color augmentation. The metrics include the area under the receiver operating characteristic curves (AUC) on the internally and externally stained and scanned slides in the paired validation set and the Pearson correlation, R, between prediction scores between the internally and externally stained and scanned slides in the paired validation set. The baseline model was initiated with ImageNet weights and used tile-level color augmentation with a set of default hyperparameters. Elements of box plot: center line, median; box limits, upper and lower quartiles; whiskers, 1.5x interquartile range. Data points in each box plot represent one model in the five-fold cross validation.

# References

1. [Germline Incidental Findings Gene List.](http://paperpile.com/b/E5HlWl/jAYx) <https://www.tempus.com/wp-content/uploads/2020/03/xT-Germline_030420.pdf>[.](http://paperpile.com/b/E5HlWl/jAYx)

2. [Green, R. C. *et al.* ACMG recommendations for reporting of incidental findings in clinical exome and genome sequencing. *Genet. Med.* **15**, 565–574 (2013).](http://paperpile.com/b/E5HlWl/bIoj)

3. [Vasen, H. F. A. *et al.* Revised guidelines for the clinical management of Lynch syndrome (HNPCC): recommendations by a group of European experts. *Gut* **62**, 812–823 (2013).](http://paperpile.com/b/E5HlWl/bcRn)

4. [Mork, M. E. *et al.* Identification of MSH2 inversion of exons 1-7 in clinical evaluation of families with suspected Lynch syndrome. *Fam. Cancer* **16**, 357–361 (2017).](http://paperpile.com/b/E5HlWl/rTPZ)
